# Polar dimming and mid-latitude brightening in a warming climate

**DOI:** 10.1093/nsr/nwag330

**Published:** 2026-05-29

**Authors:** Shichu Liu, Fengfei Song, Yuwei Wang, Lu Dong, Yu-Fan Geng, Ying Zhang

**Affiliations:** Frontier Science Center for Deep Ocean Multispheres and Earth System and Physical Oceanography Laboratory, Ocean University of China, Qingdao 266100, China; College of Oceanic and Atmospheric Sciences, Ocean University of China, Qingdao 266100, China; Key Laboratory of Physical Oceanography and Frontiers Science Center for Deep Ocean Multispheres and Earth System/Academy of the Future Ocean, Ocean University of China, Qingdao 266100, China; Frontier Science Center for Deep Ocean Multispheres and Earth System and Physical Oceanography Laboratory, Ocean University of China, Qingdao 266100, China; Laoshan Laboratory, Qingdao 266237, China; College of Oceanic and Atmospheric Sciences, Ocean University of China, Qingdao 266100, China; Frontier Science Center for Deep Ocean Multispheres and Earth System and Physical Oceanography Laboratory, Ocean University of China, Qingdao 266100, China; Laoshan Laboratory, Qingdao 266237, China; Laoshan Laboratory, Qingdao 266237, China; School of Management, Ocean University of China, Qingdao 266100, China

**Keywords:** solar radiation, seasonal cycle, climate change, climate models

## Abstract

Downward surface solar radiation (DSSR) is the dominant energy source for the Earth’s surface. It exhibits pronounced spatial heterogeneity and a strong seasonal cycle, yet how these features would evolve under global warming remains poorly understood. Here, we show a pronounced future meridional contrast in DSSR changes, with polar dimming and Northern Hemisphere (NH) mid-latitude brightening. This contrast peaks in local summer, weakening the seasonal cycle of DSSR at high latitudes with a multi-model mean of 14.6% (12.7%–17.6% for the interquartile range; same hereafter) in the Arctic, 7.0% (5.8%–8.1%) in the Antarctic, while slightly strengthening it over NH mid-latitudes by 2.1% (0.7%–3.1%) in the high-emission scenario SSP585. Under global warming, reduced clouds contribute to the mid-latitude brightening, whereas increased cloud liquid water contributes to polar dimming. Instead, increased water vapor under global warming drives the widespread clear-sky DSSR dimming. As a result, cloud-induced and clear-sky effects have comparable contributions to the polar dimming, while the cloud-induced brightening overwhelms the water-vapor-induced dimming in the NH mid-latitudes. The changes in the DSSR would alter the surface energy balance, which may exert significant influences on the polar amplification.

## Teaser

Future warming would redistribute the sunlight reaching the Earth’s surface, with less over the polar regions but more over the mid-latitudes. These changes peak in the local summer and are driven by changes in cloud properties and atmospheric water vapor.

## INTRODUCTION

Downward surface solar radiation (DSSR) provides the primary energy source for life on Earth [1]. As a fundamental component of the Earth’s surface energy budget, it regulates surface temperature, snow and ice melt, and evaporation, thereby shaping atmospheric circulation and hydrological cycles [1–7]. With the rapid expansion of solar energy deployment, DSSR has also become a critical metric for evaluating photovoltaic power potential [8–11]. Hence, it is important to illustrate the long-term changes of DSSR in a changing world.

Previous studies have established a coherent picture of the historical DSSR changes, characterized by widespread declines from the 1950s to the 1980s, followed by a subsequent increase, known as ‘global dimming and brightening’ [1,12–18]. However, our understanding of future DSSR changes remains limited, and most studies have focused on individual regions [19–24], leaving a fragmented understanding at the global scale. Although global-mean DSSR is projected to decline under high-emission scenarios and partially recover under low-emission pathways [25], such averages provide limited insight into regional-scale changes, and the associated spatial patterns remain largely unexplored [26]. Moreover, previous studies have focused mainly on the annual-mean DSSR [25,26], despite its pronounced seasonal cycle. A systematic investigation of the spatial and seasonal responses of DSSR to future global warming is urgently needed.

Regarding the factors influencing the long-term changes of DSSR, aerosol emissions have been argued to exert a dominant influence on the historical DSSR multi-decadal changes [12,15,27–31], while variations in cloud amount have substantially modulated DSSR on regional scales and shorter timescales [31–36]. Meanwhile, a recent study has shown that water vapor, through its direct absorption of shortwave radiation, contributes to global-mean DSSR changes with a magnitude comparable to that of aerosols in the historical period, and its contribution is expected to become increasingly important under future warming [25]. Besides these, surface albedo is also suggested to play a role through its interactions with aerosols [37] and clouds [38,39]. Despite these advances, the relative roles of these factors in shaping the spatial patterns and seasonal evolution of future DSSR changes remain to be quantified.

Here, using the latest Coupled Model Intercomparison Project Phase 6 (CMIP6) models, we show that DSSR changes exhibit a distinct meridional contrast, characterized by polar dimming and Northern Hemisphere (NH) mid-latitude brightening. This pattern is most prominent in the local summer, corresponding to an intensified seasonal cycle at NH mid-latitudes and a weakened cycle over the polar region. To uncover the mechanism, we decompose the DSSR response into clear-sky and cloud-induced components. Increased water vapor causes widespread clear-sky dimming and controls the spatial distribution of clear-sky DSSR by the relative change in water vapor. In response to global warming, the reduced cloud amount drives mid-latitude brightening, particularly over land, while increased cloud liquid water path (LWP) contributes to polar dimming. Overall, in the polar regions, both components act together to produce dimming, whereas in the mid-latitudes they oppose each other, with cloud-induced brightening dominating in the NH mid-latitudes. These regional and seasonal changes in the DSSR would have great implications for the polar and mid-latitude climates and applications of solar energy.

## RESULTS

### Opposite changes between mid- and high latitudes

We begin by evaluating the performance of CMIP6 models in reproducing the spatial pattern of DSSR climatology (Fig. S1). The CMIP6 models exhibit small biases in the global distribution relative to the satellite observations from Clouds and the Earth’s Radiant Energy System (CERES) [40]. This result aligns with previous studies showing that CMIP6 models represent the first generation of climate models to substantially mitigate long-standing DSSR biases [41]. We further examine the model’s ability to reproduce the DSSR trends over 2001–2024 (Fig. S2). The results indicate that the models can reasonably capture the observed trends across mid- to high-latitude regions, although with some underestimation, possibly influenced by the internal variability considering the short period (see ‘Materials and methods’ section for details). Together with measurable improvements in simulating cloud, water vapor, and shortwave absorption by water vapor [25,42,43], these advances provide confidence in using CMIP6 models to investigate the global DSSR pattern changes and the associated physical mechanisms under global warming.

**Figure 1.**
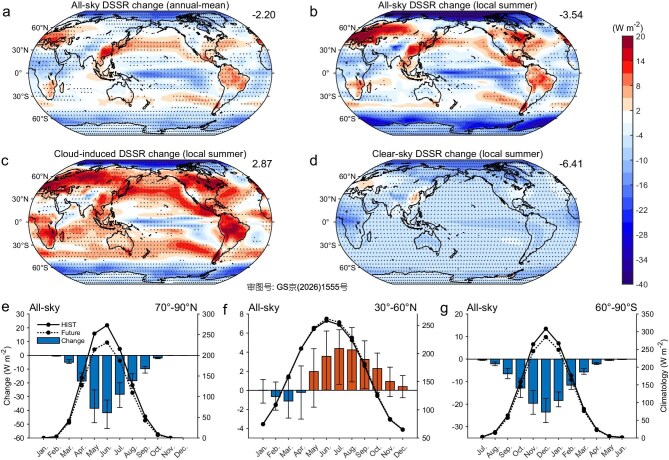
Opposite DSSR changes between mid-latitudes and polar regions. Changes in all-sky DSSR for (a) the annual-mean and (b) local summer [May–August (MJJA) for the NH and November–February (NDJF) for the SH] between 2080–2099 and 1986–2005 based on the multi-model mean (MME) of CMIP6 models. (c and d) As in panel (b), but for cloud-induced and clear-sky DSSR, respectively. Stippling in panels (a–d) indicates regions where at least 70% of the models agree on the sign of the MME change. Values in the upper right corners denote global-mean changes. MME changes in all-sky DSSR of seasonal cycle at the (e) Arctic (70°–90°N), (f) NH mid-latitudes (30°–60°N), and (g) Antarctic (60°–90°S). Solid and dashed lines indicate the historical and future climatology, respectively. Bars show the MME monthly mean changes, and error bars denote the interquartile range (25th–75th percentile) of the model ensemble. Units in panels (a–g): W m⁻². In polar regions, cloud-induced and clear-sky DSSR changes jointly cause dimming, while in mid-latitudes they act in opposite directions. The changes are most significant during the local summer, leading to a weakened seasonal cycle over polar regions but a strengthened cycle at mid-latitudes.

We analyze historical and high-emission scenario (SSP585) experiments from 36 CMIP6 models to represent present-day and future climate conditions, and quantify climate change as their difference (see ‘Materials and methods’ section). Despite a global-mean dimming as revealed by the previous work [25], the annual-mean DSSR change exhibits a pronounced spatial pattern, with most evident dimming in the Arctic and Antarctic and brightening in the NH mid-latitudes (Fig. 1a). This spatial heterogeneity is even more evident during local summer (May–August (MJJA) for NH; November–February (NDJF) for Southern Hemisphere, SH) when the climatological DSSR is the largest (Fig. 1b), and is dominated by the cloud-induced component (Fig. 1c) as the clear-sky DSSR change is quite spatially uniform (Fig. 1d).

In the polar regions during summer, both cloud-induced (defined as all-sky minus clear-sky; see ‘Materials and methods’ section) and clear-sky components contribute substantially to the projected dimming, with contributions of ∼53% and 47% for the Arctic (70°N–90°N), and 61% and 39% for the Antarctic (60°S–90°S), respectively. However, the cloud-induced and clear-sky components have opposite contributions in the mid-latitudes. Over the NH mid-latitudes (30°N–60°N), the cloud-induced effect outweighs the clear-sky change, resulting in net brightening, whereas over the SH mid-latitudes, the two effects largely cancel, yielding little net change (Fig. 1b). This may be partly because cloud-induced brightening tends to be stronger over land than ocean, while the SH has less land area, allowing clear-sky dimming to offset the cloud-induced brightening. As these changes are most evident in the local summer, the seasonal cycle of DSSR changes would be significantly suppressed over the polar regions, but enhanced over the NH mid-latitudes under global warming (Fig. 1e–g). In addition, over the NH mid-latitudes, there is a slight decrease during spring (February–April (FMA)) and a significant increase during boreal autumn (August–October (ASO)), with maximum increase 1 month later than the climatological peak (July vs June; Fig. 1f). This suggests a clear seasonal delay of DSSR over the NH mid-latitudes, which is dominated by the cloud-induced component (Fig. S3b). Quantitatively, based on the multi-model mean (MME), the seasonal cycle amplitude of DSSR would be reduced by 14.6% (the interquartile range is 12.7%–17.6%; −39.7 to −24.6 W m^−2^, the same hereafter) over the Arctic and 7.0% (5.8%–8.1%; −20.3 to −17.3 W m^−2^) over the Antarctic, while enhanced by 2.1% (0.7%–3.1%; 1.1–4.9 W m^−2^) over the NH mid-latitudes, with a phase delay of 1.1 (0.5–1.6) days. These changes are underscored by strong inter-model consensus of 100%, 100%, 86%, and 92%, respectively. The cloud-induced effect dominates the seasonal cycle changes over the Antarctic and the NH mid-latitudes, accounting 62% and 203%, respectively, whereas for the Arctic, the contributions of cloud-induced and clear-sky effects are comparable (53% vs 47%; Fig. S3). Given that the summer DSSR changes dominate both the annual-mean and the seasonal cycle changes over the mid-to-high latitudes, our subsequent analysis focuses primarily on this season to illustrate the physical reasons behind the cloud-induced and clear-sky DSSR changes.

We also examine the DSSR changes under the low-emission (SSP126) and medium-emission (SSP245) scenarios (Fig. S4). The all-sky DSSR change patterns show similar results between SSP585 (Fig. 1) and SSP126/SSP245, with all scenarios exhibiting polar dimming and NH mid-latitude brightening (Fig. S4a–c). We then focus on the high-emission scenario (SSP585) to explore the underlying mechanisms, with scenario differences discussed later.

### Relative roles of cloud amount and LWP in cloud-induced DSSR changes

**Figure 2.**
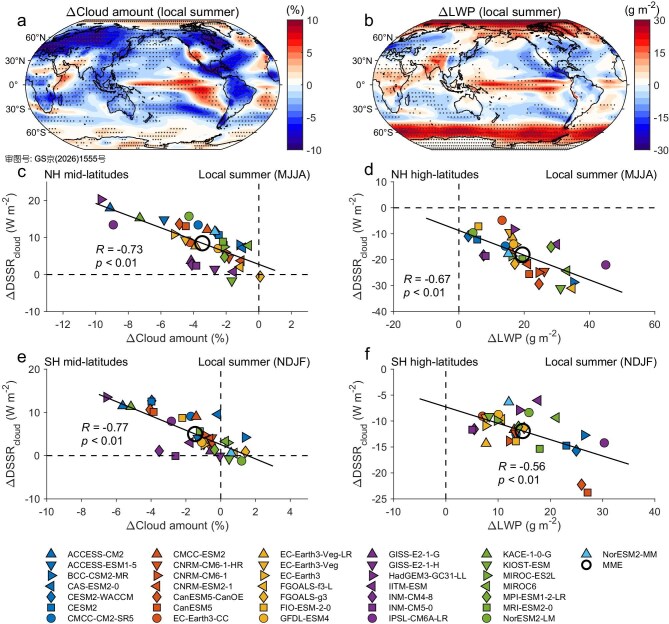
Relative roles of cloud properties in DSSR change. MME changes in (a) cloud amount (unit: %) and (b) LWP (unit: g m^−2^) of local summer between 2080–2099 and 1986–2005. Stippling indicates regions where at least 70% of the models agree on the sign of the MME change. Scatter plots of the inter-model relationship between cloud-induced DSSR changes (unit: W m^−2^) and their corresponding dominant controlling factors over (c) Arctic, (d) NH mid-latitudes, (e) Antarctic, and (f) SH high latitudes in local summer. Linear fits, correlation coefficients (*R*), and *P* values are shown. For the cloud-induced DSSR, decreased cloud amount drives brightening in mid-latitudes, while increased LWP drives dimming in high latitudes.

As the cloud-induced DSSR change underpins both polar dimming and mid-latitude brightening, we first examine the factors driving these cloud-related responses, including cloud amount and LWP [44,45]. It is found that cloud amount would decline markedly across mid-latitudes and over most land areas under global warming, with only modest increases in polar regions (Fig. 2a). In aggregate, cloud amount would decrease by 6.0% (3.2%–7.5%) and 2.4% (0.2%–4.0%) over NH and SH mid-latitudes, supported by strong inter-model consensus of 97% and 78%, respectively. The less cloud cover would allow more incoming shortwave radiation on the surface, well explaining the increased cloud-induced DSSR over the mid-latitudes. This is also well supported by strong inter-model correlations of −0.73 (*P* < 0.01) and −0.77 (*P* < 0.01) between cloud amount and cloud-induced DSSR over the NH and SH mid-latitudes, respectively (Fig. 2c and e). In contrast, LWP would increase substantially in polar regions but show only minor changes in mid-latitudes (Fig. 2b). Quantitatively, polar LWP would increase in all models by 30.7% (28.5%–60.1%) and 31.2% (23.3%–44.6%) in the Arctic and Antarctic, respectively. The increased LWP, which is linearly linked to cloud optical thickness [46], would lead to stronger reflection of shortwave radiation. Hence, the increased LWP would substantially contribute to the polar cloud-induced dimming, supported by strong inter-model correlations of −0.67 (*P* < 0.01; Fig. 2d) and −0.56 (*P* < 0.01; Fig. 2f).

Notably, unlike the cloud-induced DSSR, changes in the cloud amount and LWP do not peak in summer but are strongest in autumn over NH mid-latitudes and Arctic, respectively (Fig. S5a and d). The largest changes in the cloud-induced DSSR in summer are due to its highest sensitivity to cloud amount and LWP in this season (Fig. S5e–h), which is partly due to the larger climatological DSSR in summer, leading to greater sensitivity to cloud changes (see ‘Materials and methods’ section). In the mid-latitudes, where cloud amount is the primary contributor to the cloud-induced DSSR changes, the sensitivity of cloud-induced DSSR to cloud amount reaches −1.7 W m^−2^ %^−1^ in summer, compared with only −0.6 W m^−2^ %^−1^ in winter (Fig. S5e). In the Arctic, where LWP plays a more important role, the sensitivity to LWP is ∼−1.2 W g^−1^ in summer but nearly zero in winter (Fig. S5h). Consequently, the strongest sensitivity drives the largest cloud-induced DSSR response in summer. In addition, with comparable sensitivities in spring and autumn, the seasonal delay of DSSR over the NH mid-latitudes is instead dominated by the strongest reduction in cloud amount occurring in autumn, supported by an evident inter-model relationship of 0.82 (*P* < 0.01) between the inter-seasonal differences (ASO minus FMA) in cloud amount and cloud-induced DSSR changes (Fig. S6a). As both cloud amount and LWP are important for the cloud-induced DSSR changes, we next investigate the reasons causing the cloud amount and LWP changes in the following section.

### Potential mechanisms behind cloud amount and LWP changes

**Figure 3.**
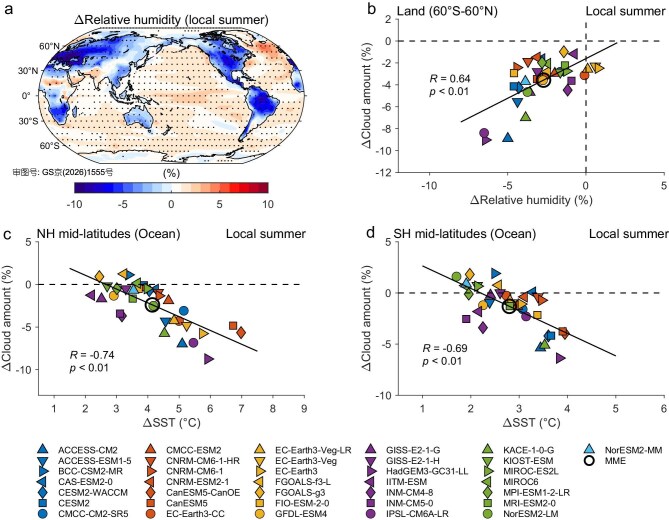
Causes of cloud amount change. (a) MME change in surface relative humidity (unit: %) of local summer between 2080–2099 and 1986–2005. Stippling indicates regions where at least 70% of the models agree on the sign of the MME change. (b) Scatter plots of the inter-model relationship between changes in humidity and cloud amount (unit: %) during local summer over land (60°S–60°N). As in panel (b), but for the changes in SST (unit: °C) and cloud amount over the (c) NH and (d) SH mid-latitude oceans, respectively. Linear fits, correlation coefficients (*R*), and *P* values are shown. Relative humidity decrease is tied to the reduced cloud amount over land, while SST increase is tied to the reduced cloud amount over ocean.

Cloud formation is inherently linked to relative humidity, with higher humidity promoting cloud development [25,47,48], as evident in observations (Fig. S7a). Over land, relative humidity decreases substantially in a warming climate, owing to limited water supply over land compared to ocean [49,50] (Fig. 3a). An inter-model relationship further indicates that a larger reduction in relative humidity is associated with a stronger decrease in cloud amount under global warming (Fig. 3b), underscored by a robust inter-model correlation coefficient of 0.64 (*P* < 0.01). Moreover, the spatial pattern of relative humidity change closely matches that of cloud amount over land, with a spatial correlation of 0.71 (*P* < 0.01).

Clouds over subtropical and mid-latitude oceans are often characterized by a prevalence of low-level clouds [51,52]. Sea surface temperature (SST) and inversion strength are considered the two main large-scale thermodynamic factors governing low-level cloud formation over ocean [53–58]. Here, higher SST is found to contribute to a reduction in cloud amount over the mid-latitude oceans (Fig. S7b). Averaged over the mid-latitude oceans, the inter-model relationships between changes in SST and cloud amount can reach −0.74 (*P* < 0.01) and −0.69 (*P* < 0.01) for NH and SH, respectively (Fig. 3c and d). It suggests that a larger increase in SST contributes to a greater decrease in cloud amount. In contrast, the increased inversion strength under global warming is generally unfavorable for cloud amount reduction, though it is important in the inter-model spread over the NH mid-latitude oceans (Fig. S8). In addition, the stronger decreased relative humidity and increased SST in autumn than in spring lead to a larger reduction in cloud amount during autumn over NH mid-latitudes, supported by inter-model correlation coefficients of 0.58 (*P* < 0.01) and −0.77 (*P* < 0.01), respectively (Fig. S6b and c). This can well explain the seasonal delay of DSSR over the NH mid-latitudes dominated by cloud amount changes.

In polar regions, where the mixed-phase clouds are common, lower-tropospheric temperature and LWP are positively correlated (Fig. S9a and b). This is because increasing temperature suppresses ice-phase microphysical processes, which reduces the conversion of liquid water to ice and precipitation, thereby enhancing cloud liquid-water retention [45]. CMIP6 models reasonably reproduce this positive relationship (Fig. S9c and d). Consequently, as ambient temperature would rise under future warming, LWP is expected to substantially increase over polar regions [59–61] (Fig. 2b).

### Global clear-sky dimming due to a wetter world

**Figure 4.**
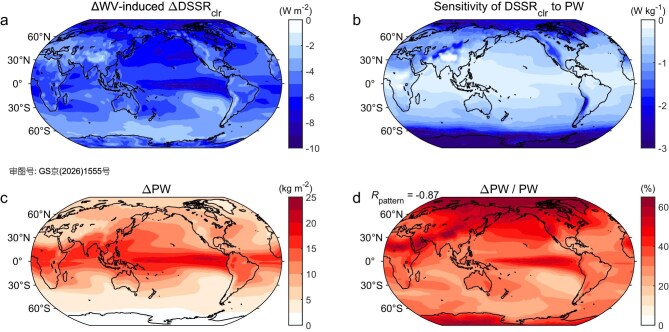
Global clear-sky dimming in a wetter world. (a) Spatial distribution of ΔWV-induced MME change in clear-sky DSSR (unit: W m^−2^) for local summer between 2080–2099 and 1986–2005. (b) Sensitivity of clear-sky DSSR to PW during the local summer for 1980–2020 (unit: W kg^−1^). As in panel (a), but for the changes in (c) PW (unit: kg m^−2^) and (d) the relative change in PW (unit: %), respectively. The number in the upper left denotes the pattern correlation between panels (a) and (d). The ΔWV-induced change is computed using the CESM-CAM5 radiative kernel. The sensitivity calculation is based on the historical greenhouse-gas-only (hist-GHG) experiment of 11 CMIP6 models (see ‘Materials and methods’ section). The clear-sky dimming results from increased water vapor, and its spatial pattern is largely governed by the relative changes in water vapor.

The clear-sky DSSR changes contribute substantially to the polar dimming (∼47% for the Arctic and 39% for the Antarctic), so it is important to illustrate the drivers beneath the changes. The increasing atmospheric water vapor under global warming would cause clear-sky dimming, exerting a competing effect with the brightening associated with projected aerosol reductions (Fig. S10). We further estimate the radiative effect of the change in water vapor (ΔWV) on the clear-sky DSSR by applying a surface radiative kernel (see ‘Materials and methods’ section). The ΔWV-induced clear-sky DSSR exhibits a spatial pattern that closely matches the simulated clear-sky DSSR change, with a pattern correlation of 0.71 (*P* < 0.01; Fig. 4a vs Fig. 1d), suggesting that ΔWV plays a major role in the clear-sky DSSR change.

To explain the summer peak in clear-sky DSSR changes, we examine the seasonal behavior of the column water vapor (precipitable water, PW) changes and the sensitivity of clear-sky DSSR to PW. Taking the NH as an example, PW in the mid- and high latitudes exhibits its largest increases in summer, with 9.0 and 6.3 kg m^−2^, respectively, approximately twice those in winter (Fig. S11a and b). Over the NH mid-latitudes, the sensitivity of clear-sky DSSR to PW is comparable between summer and winter (−0.87 vs −0.74 W m^−2^ kg^−1^; Fig. S11c), indicating that the summer maximum in clear-sky DSSR changes is primarily driven by the largest PW increases. In contrast, in the Arctic, clear-sky DSSR exhibits the strongest sensitivity to PW in summer (Fig. S11d), such that the summer peak arises from the combined effects of enhanced PW increases and heightened sensitivity. Similar situations also hold for the SH.

We next turn to the spatial structure of ΔWV-induced clear-sky DSSR changes, which exhibits pronounced dimming in both polar and tropical regions (Fig. 4a). Clear-sky DSSR is most sensitive to PW in the polar regions, possibly related to low solar elevation and extended summer daylight, with sensitivities nearly an order of magnitude larger than in the tropics (Fig. 4b). In contrast, the largest PW increase occurs in the tropics, while polar increases are only ∼20% of tropical values (Fig. 4c). The spatial pattern of ΔWV-induced clear-sky DSSR responses thus reflects a combination between the sensitivity of PW and the magnitude of PW increases. It also highlights the need for a more representative metric that better captures the spatial distribution of clear-sky DSSR responses to PW changes. Here, it is found that ΔWV-induced clear-sky DSSR changes depend primarily on the relative (ΔPW/PW) change rather than the absolute change. Indeed, the spatial pattern of relative PW change strongly resembles the ΔWV-induced dimming, with a pattern correlation of −0.87 (*P* < 0.01; Fig. 4a vs Fig. 4d). This behavior can be explained by the Beer–Lambert law, which states the changes in clear-sky DSSR scale with the relative change in PW (see ‘Materials and methods’ section). As a result, regions with a larger increase generally experience stronger clear-sky dimming, except for some high-elevation areas such as the Tibetan Plateau.

## DISCUSSION AND CONCLUSIONS

In this study, we show pronounced meridional and seasonal contrasts in the DSSR changes based on CMIP6 models under a high-emission scenario (SSP585), with dimming over the polar regions and brightening across the NH mid-latitudes. These changes are most pronounced during the local summer, leading to a weakening of the seasonal cycle in the polar regions and an amplified seasonal cycle in the NH mid-latitudes. Decomposing DSSR changes into clear-sky and cloud-induced components shows that increased water vapor drives clear-sky dimming across all latitudes, with its spatial pattern closely following relative changes in water vapor. In the mid-latitudes, decreased relative humidity over land and increased SST over ocean drive cloud amount reduction, resulting in brightening, particularly over land. In polar regions, warming increases LWP, leading to thicker clouds and consequent dimming. Overall, cloud changes and increased water vapor act together to drive the polar dimming, but have opposing effects in the mid-latitudes, with the cloud-induced brightening outweighing the clear-sky dimming over NH and yielding an insignificant net change over SH. In addition, polar dimming tends to scale with emission intensity by analyzing different warming scenarios, while the magnitude of NH mid-latitude brightening remains relatively consistent across all scenarios (Fig. S4d–f). The strength of cloud-induced brightening in the mid-latitudes also tends to scale with emission intensity. However, given that aerosol reductions are more pronounced in the low/medium emission scenarios (SSP126/SSP245) [25], this is associated with clear-sky brightening in these regions, partly compensating for the relatively weaker cloud-induced brightening. As a result, NH mid-latitude brightening remains largely consistent across all three scenarios.

The Arctic is a key region in climate change research and exhibits the most pronounced DSSR response, with a projected decrease of 15.2% (11.4%–19.1%) during May–June by the end of the century under SSP585. The DSSR changes in the Arctic have great implications, including alterations in the surface energy balance (Fig. S12). It suggests that DSSR dimming offsets a substantial portion of the net increase in surface heat flux (which is dominated by the increase in absorbed shortwave radiation associated with surface albedo reduction), on the order of ∼50%, during local summer in the Arctic (Fig. S12i). Inter-model relationships further reveal strong coupling between changes in DSSR and upward surface solar radiation [*R* = −0.70 (*P* < 0.01)], as well as upward and downward surface longwave radiation [*R* = 0.50 and −0.58 (*P* < 0.01), respectively; Fig. S13]. These indicate that even though other surface energy fluxes may offset or reinforce DSSR changes, the overall surface energy balance is altered, implying a corresponding adjustment of the climate system. Quantifying the contribution of DSSR changes to the surface energy budget, as well as their interactions with dynamical processes, remains an important topic for future research. Ultimately, understanding the role of Arctic dimming within the broader context of surface energy balance is crucial for future Arctic climate change research.

We also examine the relationships between projected surface temperature changes and DSSR changes (Fig. S14). There is a significant negative inter-model relationship between projected Arctic surface temperature warming and DSSR dimming [Fig. S14a and b; *R* = −0.57 (*P* < 0.01) for annual mean and *R* = −0.47 (*P* < 0.01) for local summer]. In contrast, over the NH mid-latitudes, DSSR brightening is positively correlated with surface warming [Fig. S14c and d; *R* = 0.40 (*P* = 0.02) for annual mean; *R* = 0.58 (*P* < 0.01) for local summer]. These opposite relationships imply that DSSR changes act as negative radiative feedback on Arctic warming, while reinforcing surface warming over the NH mid-latitudes.

In this study, our results highlight the meridionally and seasonally dependent DSSR response to global warming, with far-reaching implications for surface energy balance, hydrological processes, cryosphere dynamics, and solar power generation that merit further investigation. Regarding the mechanisms, we focus on the respective roles of cloud-property changes and water-vapor changes in driving cloud-induced and clear-sky DSSR changes. As polar regions experience particularly rapid warming, snow and ice are projected to decline substantially, leading to pronounced surface albedo reduction. Recent studies suggest that reduced polar albedo may enhance cloud-induced shortwave cooling [38,39] and also affect clear-sky radiation by modulating aerosol radiative effects [37], thereby influencing DSSR. These findings imply that polar albedo reduction may contribute to both cloud-induced and clear-sky dimming through multiple pathways, and its role in polar DSSR dimming warrants future quantification.

## MATERIALS AND METHODS

### Model simulations and observational data

To examine future changes in DSSR and their underlying mechanisms under global warming, we analyze 36 CMIP6 model simulations [62] from the historical and SSP585 experiments. Historical simulations are driven by observed anthropogenic and natural forcings through 2014, followed by a high-emission SSP585 scenario through 2100. The SSP126 low-emission and SSP245 medium-emission scenarios are also used to examine the robustness of our results. We take 1986–2005 from the historical experiment as the present-day climate and 2080–2099 from the SSP585 scenario as the future climate. In this study, ‘change’ is defined as the difference between the climatological means of these two periods (2080–2099 minus 1986–2005). We define local summer as May–August (NH) and November–February (SH) instead of the conventional June–August (JJA)/December–February (DJF) definition, as DSSR peaks earlier and values in May/November are comparable to or exceed those in late summer (August/February; Fig. 1). Using JJA/DJF yields similar results, indicating little sensitivity to this definition. To examine the sensitivity of clear-sky DSSR to PW, we also analyze 11 CMIP6 simulations from the hist-GHG experiment, which is driven by historical greenhouse gas forcing until 2020. We use one realization from each model to ensure equal weighting in the multi-model mean. Prior to analysis, all model outputs are interpolated onto a common 2.5° × 2.5° grid.

Monthly model outputs of downward surface shortwave radiation (all-sky DSSR), downward surface clear-sky shortwave radiation (clear-sky DSSR), upward surface all-sky shortwave radiation, downward and upward surface longwave radiation, surface latent and sensible heat flux, total cloud cover percentage, ice water path, condensed water path, near-surface relative humidity, surface air pressure, surface temperature, ambient aerosol optical thickness at 500 nm, air temperature, and specific humidity are utilized. The LWP is derived by subtracting the ice water path from the condensed water path. Some variables are not available in all models and the detailed information is provided in Table S1.

We use satellite observations from the CERES [40] Energy Balanced and Filled dataset to evaluate CMIP6 DSSR climatology and recent trends from 2001 to 2024. The CERES dataset provides observation-based radiative fluxes derived from top-of-atmosphere measurements since 2001. We also use monthly outputs from the European Centre for Medium-Range Weather Forecasts Reanalysis v5 [63] to examine the relationships between mid‐latitude cloud amount, relative humidity, and SST, and to assess the performance of CMIP6 models in simulating the relationship between polar LWP and lower‐tropospheric temperature.

### Evaluation of CMIP6 simulations of DSSR

To assess the reliability of CMIP6 DSSR simulations, we evaluate their climatology and recent trends against CERES observations over 2001–2024. The evaluation is based on the historical and SSP585 experiments from CMIP6. We first examine the spatial distribution of DSSR climatology (Fig. S1) and find that CMIP6 models generally capture the observed large-scale patterns with relatively small biases. For the trend evaluation, the results indicate that CMIP6 models reasonably reproduce the observed DSSR trends over the key regions examined in this study, including Arctic dimming (observations vs MME: −5.7 vs −3.0 W m^−2^ per decade; all trends refer to local summer), NH mid-latitude brightening (1.3 vs 0.6 W m^−2^ per decade), and Antarctic dimming (−2.4 vs −1.3 W m^−2^ per decade), although the magnitudes are generally underestimated (Fig. S2). The underestimation is possibly influenced by the internal variability due to the short period considered here. Similarly, a recent work [25] has shown that CMIP6 models can reasonably reproduce long-term DSSR trends since 1959 over regions such as China, India, the eastern USA, and western Europe, based on comparisons with station data from the Global Energy Balance Archive [64]. Taken together, these results provide confidence in using CMIP6 simulations to analyze future DSSR changes.

### Cloud radiative effect

In this study, we define the cloud radiative effect on the DSSR (cloud-induced DSSR) as the difference between all-sky DSSR and clear-sky DSSR:


(1) \begin{eqnarray*}
\Delta {\textit{DSS}}{{\mathrm{R}}}_{{\mathrm{cloud}}} = \Delta {\textit{DSS}}{{\mathrm{R}}}_{{\mathrm{all}}} - {\textit{DSS}}{{\textit{R}}}_{{\mathrm{clr}}}.
\end{eqnarray*}

Here, all-sky DSSR is computed with clouds included, whereas clear-sky DSSR is obtained under identical atmospheric conditions but without cloud–radiation interactions. Under this framework, the cloud-induced DSSR is diagnosed as the residual between all-sky and clear-sky conditions, implicitly assuming a linear decomposition of radiative contributions. This approach does not account for the covariance between clouds and the atmospheric state [e.g. 65]. Despite this, it provides a useful way to quantify the cloud-induced radiative contribution, which is widely adopted in previous studies [e.g. 66,67].

### Sensitivity of DSSR to physical drivers

The sensitivity of DSSR to a given factor is defined as the regression coefficient of monthly DSSR anomalies against the corresponding anomalies in that factor. For each model, the sensitivity is estimated separately for each calendar month using the monthly data. Specifically, for a factor *X* (e.g. cloud amount, LWP, or PW), the sensitivity *S* is obtained from the linear regression:


(2) \begin{eqnarray*}
\Delta {\textit{DSS}}{{\mathrm{R}}}_m (y) = {S}_{X,m}\cdot\Delta {X}_m (y) + {\varepsilon }_m (y),\end
{eqnarray}$$

where *m* denotes calendar month, *y* denotes year, and *S_X,m_* represents the change in DSSR associated with a unit change in *X* for month *m*. Sensitivities are diagnosed separately for clear-sky DSSR and cloud-induced DSSR. Prior to the regression analysis, all variables are linearly detrended to remove long-term trends and isolate the inter-annual variability.

Because these sensitivities depend on the prevailing radiative conditions, they are not fixed parameters and vary with season and region. In particular, sensitivities tend to peak in summer, when the climatological DSSR is the largest, making DSSR more responsive to perturbations in atmospheric properties, whereas they are the weakest in winter. Accordingly, the seasonal variations of sensitivity broadly follow the climatological annual cycle of DSSR (e.g. Fig. S5e–h). In contrast, the sensitivity of clear-sky DSSR to PW over the mid-latitudes remains relatively uniform throughout the year (Fig. S11c). This reflects the competing effects of solar radiation and atmospheric optical path length: stronger incoming solar radiation in summer tends to enhance the sensitivity, while the shorter optical path reduces the absorption efficiency of water vapor, and vice versa in winter. As a result, these opposing effects lead to a relatively weak seasonal dependence of PW sensitivity.

### Radiative kernel

We apply a surface clear-sky shortwave moisture kernel (*K*) derived from CAM5 to simply quantify the influence of water-vapor change ($\Delta {\mathrm{WV}}$) on the clear-sky DSSR dimming [68]. The ΔWV-induced SSR ($\Delta {\textit{SS}}{{\textit{R}}}_{{\mathrm{WV}}}$) is calculated as


(3) \begin{eqnarray*}
\Delta {\textit{SS}}{{\textit{R}}}_{{\mathrm{WV}}} = \ \left\langle {K\cdot\Delta {\mathrm{WV}}} \right\rangle,
\end{eqnarray*}

where the angle bracket denotes vertical integration between the surface and tropopause; the $\Delta {\textit{SS}}{{\textit{R}}}_{{\mathrm{WV}}}$ is the ΔWV-induced surface net solar radiation, which is closely tied to the DSSR via the surface albedo $\alpha $: $\Delta {\textit{SS}}{{\textit{R}}}_{{\mathrm{WV}}} = ( {1 - \alpha } ) \cdot \,\Delta {\textit{DSS}}{{\mathrm{R}}}_{{\mathrm{WV}}}$. Hence, the DSSR changes can be written as


(4) \begin{eqnarray*}
\Delta {\textit{DSS}}{{\textit{R}}}_{{\mathrm{WV}}} = \Delta {\textit{SS}}{{\textit{R}}}_{{\mathrm{WV}}}\cdot{\left( {1 - \alpha } \right)}^{ - 1}.
\end{eqnarray*}

The $\alpha $ is calculated as the ratio of upward to downward shortwave radiation at the surface. It is important to note that the kernel is constructed from a historical climate state, and as such, it is state-dependent [68,69]. Moreover, since the kernel is a linear method, it may not fully capture interactions between variables, such as the nonlinear relationship between water vapor and sea ice [70]. Therefore, the kernel-derived response calculated here is a rough approximation. Future research requires more sophisticated radiative transfer models to provide finer quantification. Given these limitations, this study focuses on comparing spatial distributions of the kernel-derived response and climate model outputs, rather than on absolute values.

### Beer–Lambert law

We refer to the Beer–Lambert law to understand why the spatial pattern of ΔWV-induced clear-sky dimming follows the relative change in PW. The Beer–Lambert law describes the attenuation of direct radiation as it travels through an absorbing medium. For clear-sky conditions, the clear-sky DSSR (*S*) at the surface can be written as


(5) \begin{eqnarray*}
S = \ {S}_0\cdot{e}^{ - \tau },
\end{eqnarray*}

where ${S}_0$ is the incoming solar radiation at the top of the atmosphere, and $\tau $ is the optical depth. Assuming that water-vapor absorption dominates and is proportional to the PW, we express $\tau = k\cdot{{\rm PW}}\cdot m$, where *k* is the absorption coefficient and *m* is the air-mass factor, representing the relative path length of sunlight through the atmosphere. Thus, the change in ΔWV-induced clear-sky DSSR can be written as


(6) \begin{eqnarray*}
\Delta S &=& {S}_0\left( {{e}^{ - k\cdot\left( {{\rm {PW}} + \Delta {\rm {PW}}} \right)\cdot m} - {e}^{ - k\cdot{\rm {PW}}\cdot m}} \right),\\
\Delta S &=& S\left( {{e}^{ - k\cdot\Delta {\rm {PW}}\cdot m} - 1} \right),\\
\Delta S &=& S\left( {{e}^{ - \tau \cdot r} - 1} \right),
\end{eqnarray*}

where *r* is the relative change in PW (ΔPW/PW). For small changes in PW, a first-order Taylor expansion gives the change in *S* as


(7) \begin{eqnarray*}
\Delta S = - S\cdot\tau \cdot r.
\end{eqnarray*}

This indicates that the change in ΔWV-induced clear-sky DSSR radiation scales linearly with the relative change in PW. The spatial pattern of $\Delta S$ approximately follows the spatial distribution of *r* (Fig. 4a vs Fig. 4d), suggesting the spatial variability of the product $S\cdot\tau $ is relatively small.

## Supplementary Material

nwag330_Supplemental_File

## Data Availability

All data used in this study are publicly available. The CMIP6 data is available at https://aims2.llnl.gov/search/. ECMWF Reanalysis v5 is available at https://cds.climate.copernicus.eu/datasets/reanalysis-era5-single-levels-monthly-means. CERES satellite observation is available at https://terra.nasa.gov/data/ceres-data. CESM-CAM5 radiative kernels are available at https://zenodo.org/records/997902. Codes for reproducing the results are available from the corresponding author on request.

## References

[1] Wild M . Global dimming and brightening: a review. J Geophys Res 2009; 114: D00D16. 10.1029/2008JD011470

[2] Wang K, Dickinson RE. Contribution of solar radiation to decadal temperature variability over land. Proc Natl Acad Sci USA 2013; 110: 14877–82. 10.1073/pnas.1311433110 23980136 PMC3773731

[3] Giesen RH, van den Broeke MR, Oerlemans J et al. Surface energy balance in the ablation zone of Midtdalsbreen, a glacier in southern Norway: interannual variability and the effect of clouds. J Geophys Res 2008; 113: D21111. 10.1029/2008JD010390

[4] Roderick ML, Farquhar GD. The cause of decreased pan evaporation over the past 50 years. Science 2002; 298: 1410–1. 10.1126/science.1075390-a 12434057

[5] Wild M, Ohmura A, Gilgen H et al. On the consistency of trends in radiation and temperature records and implications for the global hydrological cycle. Geophys Res Lett 2004; 31: L11201. 10.1029/2003GL019188

[6] Ramanathan V, Crutzen PJ, Kiehl JT et al. Aerosols, climate, and the hydrological cycle. Science 2001; 294: 2119–24. 10.1126/science.1064034 11739947

[7] Wang Y, Meili N, Fatichi S. Ecohydrological responses to solar radiation changes. Hydrol Earth Syst Sci 2025; 29: 381–96. 10.5194/hess-29-381-2025

[8] Polo J, Wilbert S, Ruiz-Arias JA et al. Preliminary survey on site-adaptation techniques for satellite-derived and reanalysis solar radiation datasets. Sol Energy 2016; 132: 25–37. 10.1016/j.solener.2016.03.001

[9] Lei Y, Wang Z, Wang D et al. Co-benefits of carbon neutrality in enhancing and stabilizing solar and wind energy. Nat Clim Chang 2023; 13: 693–700. 10.1038/s41558-023-01692-7

[10] Tong D, Farnham DJ, Duan L et al. Geophysical constraints on the reliability of solar and wind power worldwide. Nat Commun 2021; 12: 6146. 10.1038/s41467-021-26355-z 34686663 PMC8536784

[11] Gernaat DEHJ, de Boer HS, Daioglou V et al. Climate change impacts on renewable energy supply. Nat Clim Chang 2021; 11: 119–25. 10.1038/s41558-020-00949-9

[12] Wang Z, Wang C, Yang S et al. Evaluation of surface solar radiation trends over China since the 1960s in the CMIP6 models and potential impact of aerosol emissions. Atmos Res 2022; 268: 105991. 10.1016/j.atmosres.2021.105991

[13] Pinker RT, Zhang B, Dutton EG. Do satellites detect trends in surface solar radiation? Science 2005; 308: 850–4. 10.1126/science.1103159 15879215

[14] Wild M, Gilgen H, Roesch A et al. From dimming to brightening: decadal changes in solar radiation at Earth’s surface. Science 2005; 308: 847–50. 10.1126/science.1103215 15879214

[15] Wild M . Enlightening global dimming and brightening. Bull Amer Meteor Soc 2012; 93: 27–37. 10.1175/BAMS-D-11-00074.1

[16] Schwarz M, Folini D, Yang S et al. Changes in atmospheric shortwave absorption as important driver of dimming and brightening. Nat Geosci 2020; 13: 110–5. 10.1038/s41561-019-0528-y

[17] Yuan M, Leirvik T, Wild M. Global trends in downward surface solar radiation from spatial interpolated ground observations during 1961–2019. J Clim 2021: 1–56. 10.1175/JCLI-D-21-0165.1

[18] Stanhill G, Cohen S. Global dimming: a review of the evidence for a widespread and significant reduction in global radiation with discussion of its probable causes and possible agricultural consequences. Agric For Meteorol 2001; 107: 255–78. 10.1016/S0168-1923(00)00241-0

[19] Gutiérrez C, Somot S, Nabat P et al. Future evolution of surface solar radiation and photovoltaic potential in Europe: investigating the role of aerosols. Environ Res Lett 2020; 15: 034035. 10.1088/1748-9326/ab6666

[20] Räisänen P, Ruosteenoja K. Seasonal changes in solar radiation and relative humidity in Europe in response to global warming. J Clim 2013; 26: 2467–81. 10.1175/JCLI-D-12-00007.1

[21] Chen L . Uncertainties in solar radiation assessment in the United States using climate models. Clim Dyn 2020; 56: 665–78. 10.1007/s00382-020-05498-7

[22] Pan Z, Segal M, Arritt RW et al. On the potential change in solar radiation over the US due to increases of atmospheric greenhouse gases. Renew Energy 2004; 29: 1923–8. 10.1016/j.renene.2003.11.013

[23] He Y, Yang K, Wild M et al. Constrained future brightening of solar radiation and its implication for China’s solar power. Natl Sci Rev 2023; 10: nwac242. 10.1093/nsr/nwac242 36654914 PMC9840459

[24] Ruosteenoja K, Räisänen P, Devraj S et al. Future changes in incident surface solar radiation and contributing factors in India in CMIP5 climate model simulations. J Appl Meteorol Climatol 2019; 58: 19–35. 10.1175/JAMC-D-18-0013.1

[25] Song F, Mao Y, Liu S et al. A long-term decline in downward surface solar radiation. Natl Sci Rev 2025; 12: nwaf007. 10.1093/nsr/nwaf007 39989914 PMC11846084

[26] Wild M, Folini D, Henschel F et al. Projections of long-term changes in solar radiation based on CMIP5 climate models and their influence on energy yields of photovoltaic systems. Sol Energy 2015; 116: 12–24. 10.1016/j.solener.2015.03.039

[27] Julsrud IR, Storelvmo T, Schulz M et al. Disentangling aerosol and cloud effects on dimming and brightening in observations and CMIP6. J Geophys Res Atmos 2022; 127: e2021JD035476. 10.1029/2021JD035476

[28] Wang KC, Dickinson RE, Wild M et al. Atmospheric impacts on climatic variability of surface incident solar radiation. Atmos Chem Phys 2012; 12: 9581–92. 10.5194/acp-12-9581-2012

[29] Streets DG, Wu Y, Chin M. Two-decadal aerosol trends as a likely explanation of the global dimming/brightening transition. Geophys Res Lett 2006; 33: L15806. 10.1029/2006GL026471

[30] Romanou A, Liepert B, Schmidt GA et al. 20th century changes in surface solar irradiance in simulations and observations. Geophys Res Lett 2007; 34: L05713. 10.1029/2006GL028356

[31] Yang S, Wang XL, Wild M. Causes of dimming and brightening in China inferred from homogenized daily clear-sky and all-sky in situ surface solar radiation records (1958–2016). J Clim 2019; 32: 5901–13. 10.1175/JCLI-D-18-0666.1

[32] Stanhill G, Achiman O, Rosa R et al. The cause of solar dimming and brightening at the Earth’s surface during the last half century: evidence from measurements of sunshine duration. J Geophys Res Atmos 2014; 119: 10902–11. 10.1002/2013JD021308

[33] Padma Kumari B, Goswami BN. Seminal role of clouds on solar dimming over the Indian monsoon region. Geophys Res Lett 2010; 37: L06703. 10.1029/2009GL042133

[34] Long CN, Dutton EG, Augustine JA et al. Significant decadal brightening of downwelling shortwave in the continental United States. J Geophys Res 2009; 114: D00D06. 10.1029/2008JD011263

[35] Jahani B, Dinpashoh Y, Wild M. Dimming in Iran since the 2000s and the potential underlying causes. Int J Climatol 2017; 38: 1543–59. 10.1002/joc.5265

[36] Augustine JA, Dutton EG. Variability of the surface radiation budget over the United States from 1996 through 2011 from high-quality measurements. J Geophys Res Atmos 2013; 118: 43–53. 10.1029/2012JD018551

[37] Chen A, Zhao C, Zhang H et al. Surface albedo regulates aerosol direct climate effect. Nat Commun 2024; 15: 7816. 10.1038/s41467-024-52255-z 39242629 PMC11379713

[38] Chen A, Zhao C, Zhang H et al. Weakened snow and ice melting by enhanced cloud short-wave cooling effect in the Arctic. Natl Sci Rev 2025; 12: nwaf116. 10.1093/nsr/nwaf116 40330045 PMC12051870

[39] Zhang H, Zhao C, Li J et al. Shortwave cloud warming effect observed over highly reflective Greenland. Sci Bull 2025; 70: 951–9. 10.1016/j.scib.2025.01.027 39922781

[40] Loeb NG, Doelling DR, Wang H et al. Clouds and the Earth’s Radiant Energy System (CERES) Energy Balanced and Filled (EBAF) Top-of-Atmosphere (TOA) Edition-4.0 data product. J Clim 2018; 31: 895–918. 10.1175/JCLI-D-17-0208.1

[41] Wild M . The global energy balance as represented in CMIP6 climate models. Clim Dyn 2020; 55: 553–77. 10.1007/s00382-020-05282-7 32704207 PMC7366598

[42] Jiang JH, Su H, Wu L et al. Improvements in cloud and water vapor simulations over the tropical oceans in CMIP6 compared to CMIP5. Earth Space Sci 2021; 8: e2020EA001520. 10.1029/2020EA001520

[43] Zheng X, Tao C, Zhang C et al. Assessment of CMIP5 and CMIP6 AMIP simulated clouds and surface shortwave radiation using ARM observations over different climate regions. J Clim 2023; 36: 8475–95. 10.1175/JCLI-D-23-0247.1

[44] Zelinka MD, Klein SA, Hartmann DL. Computing and partitioning cloud feedbacks using cloud property histograms. Part II: attribution to changes in cloud amount, altitude, and optical depth. J Clim 2012; 25: 3736–54. 10.1175/JCLI-D-11-00249.1

[45] Ceppi P, Brient F, Zelinka MD et al. Cloud feedback mechanisms and their representation in global climate models. WIREs Clim Change 2017; 8: e465. 10.1002/wcc.465

[46] Stephens GL . Radiation profiles in extended water clouds. II: parameterization schemes. J Atmos Sci 1978; 35: 2123–32. 10.1175/1520-0469(1978)035<2123:RPIEWC>2.0.CO;2

[47] Walcek CJ . Cloud cover and its relationship to relative humidity during a springtime midlatitude cyclone. Mon Weather Rev 1994; 122: 1021–35. 10.1175/1520-0493(1994)122<1021:CCAIRT>2.0.CO;2

[48] Zhang M, Bretherton C. Mechanisms of low cloud–climate feedback in idealized single-column simulations with the community atmospheric model, version 3 (CAM3). J Clim 2008; 21: 4859–78. 10.1175/2008JCLI2237.1

[49] O’Gorman PA, Muller CJ. How closely do changes in surface and column water vapor follow Clausius–Clapeyron scaling in climate change simulations? Environ Res Lett 2010; 5: 025207. 10.1088/1748-9326/5/2/025207

[50] Byrne MP, O’Gorman PA. Understanding decreases in land relative humidity with global warming: conceptual model and GCM simulations. J Clim 2016; 29: 9045–61. 10.1175/JCLI-D-16-0351.1

[51] Hartmann DL, Ockert-Bell ME, Michelsen ML. The effect of cloud type on Earth’s energy balance: global analysis. J Clim 1992; 5: 1281–304. 10.1175/1520-0442(1992)005<1281:TEOCTO>2.0.CO;2

[52] Klein SA, Hartmann DL. The seasonal cycle of low stratiform clouds. J Clim 1993; 6: 1587–606. 10.1175/1520-0442(1993)006<1587:TSCOLS>2.0.CO;2

[53] Qu X, Hall A, Klein SA et al. On the spread of changes in marine low cloud cover in climate model simulations of the 21st century. Clim Dyn 2013; 42: 2603–26. 10.1007/s00382-013-1945-z

[54] Bretherton CS, Wyant MC. Moisture transport, lower-tropospheric stability, and decoupling of cloud-topped boundary layers. J Atmos Sci 1997; 54: 148–67. 10.1175/1520-0469(1997)054<0148:MTLTSA>2.0.CO;2

[55] Brient F, Bony S. Interpretation of the positive low-cloud feedback predicted by a climate model under global warming. Clim Dyn 2012; 40: 2415–31. 10.1007/s00382-011-1279-7

[56] Rieck M, Nuijens L, Stevens B. Marine boundary layer cloud feedbacks in a constant relative humidity atmosphere. J Atmos Sci 2012; 69: 2538–50. 10.1175/JAS-D-11-0203.1

[57] Chung D, Teixeira J. A simple model for stratocumulus to shallow cumulus cloud transitions. J Clim 2012; 25: 2547–54. 10.1175/JCLI-D-11-00105.1

[58] Wood R, Bretherton CS. On the relationship between stratiform low cloud cover and lower-tropospheric stability. J Clim 2006; 19: 6425–32. 10.1175/JCLI3988.1

[59] McCoy DT, Hartmann DL, Zelinka MD et al. Mixed-phase cloud physics and Southern Ocean cloud feedback in climate models. J Geophys Res Atmos 2015; 120: 9539–54. 10.1002/2015JD023603

[60] Ceppi P, Hartmann DL, Webb MJ. Mechanisms of the negative shortwave cloud feedback in middle to high latitudes. J Clim 2016; 29: 139–57. 10.1175/JCLI-D-15-0327.1

[61] Tsushima Y, Emori S, Ogura T et al. Importance of the mixed-phase cloud distribution in the control climate for assessing the response of clouds to carbon dioxide increase: a multi-model study. Clim Dyn 2006; 27: 113–26. 10.1007/s00382-006-0127-7

[62] Eyring V, Bony S, Meehl GA et al. Overview of the coupled model intercomparison project phase 6 (CMIP6) experimental design and organization. Geosci Model Dev 2016; 9: 1937–58. 10.5194/gmd-9-1937-2016

[63] Hersbach H, Bell B, Berrisford P et al. The ERA5 global reanalysis. Quart J Royal Meteorol Soc 2020; 146: 1999–2049. 10.1002/qj.3803

[64] Wild M, Ohmura A, Schär C et al. The global energy balance archive (GEBA) version 2017: a database for worldwide measured surface energy fluxes. Earth Syst Sci Data 2017; 9: 601–13. 10.5194/essd-9-601-2017

[65] Soden BJ, Held IM, Colman R et al. Quantifying climate feedbacks using radiative kernels. J Clim 2008; 21: 3504–20. 10.1175/2007JCLI2110.1

[66] Lubis SW, Harrop BE, Lu J et al. Cloud radiative effects significantly increase wintertime atmospheric blocking in the Euro-Atlantic sector. Nat Commun 2025; 16: 9763. 10.1038/s41467-025-64672-9 41193422 PMC12589630

[67] Voigt A, North S, Gasparini B et al. Atmospheric cloud-radiative heating in CMIP6 and observations and its response to surface warming. Atmos Chem Phys 2024; 24: 9749–75. 10.5194/acp-24-9749-2024

[68] Pendergrass AG, Conley A, Vitt FM. Surface and top-of-atmosphere radiative feedback kernels for CESM-CAM5. Earth Syst Sci Data 2018; 10: 317–24. 10.5194/essd-10-317-2018

[69] Huang H, Huang Y. Radiative sensitivity quantified by a new set of radiation flux kernels based on the ECMWF Reanalysis v5 (ERA5). Earth Syst Sci Data 2023; 15: 3001–21. 10.5194/essd-15-3001-2023

[70] Huang Y, Huang H, Shakirova A. The nonlinear radiative feedback effects in the Arctic warming. Front Earth Sci 2021; 9: 693779. 10.3389/feart.2021.693779

