# Supplementary material for: Polar dimming and mid-latitude brightening in a warming climate
Source: Natl Sci Rev. 2026 May 29;13(13):nwag330. doi: 10.1093/nsr/nwag330 (PMC13348249; doi:10.1093/nsr/nwag330)
Supplement: nwag330_Supplemental_File [file nwag330_supplemental_file.pdf]

Supplementary materials:

Polar dimming and mid-latitude brightening in a warming climate

Shichu Liu[1,3,4], Fengfei Song*[1,2], Yuwei Wang[3], Lu Dong[1,2], Yu-Fan Geng[2], Ying Zhang[5]

1. Frontier Science Center for Deep Ocean Multispheres and Earth System and Physical Oceanography Laboratory, Ocean University of China, Qingdao 266100, China

2. Laoshan Laboratory, Qingdao 266237, China

3. College of Oceanic and Atmospheric Sciences, Ocean University of China, Qingdao 266100, China

4. Key Laboratory of Physical Oceanography and Frontiers Science Center for Deep Ocean Multispheres and Earth System/Academy of the Future Ocean, Ocean University of China, Qingdao 266100, China

5. School of Management, Ocean University of China, Qingdao 266100, China

*** Corresponding authors:**

Fengfei Song (songfengfei@ouc.edu.cn)

There are 1 table and 14 figures in this appendix.

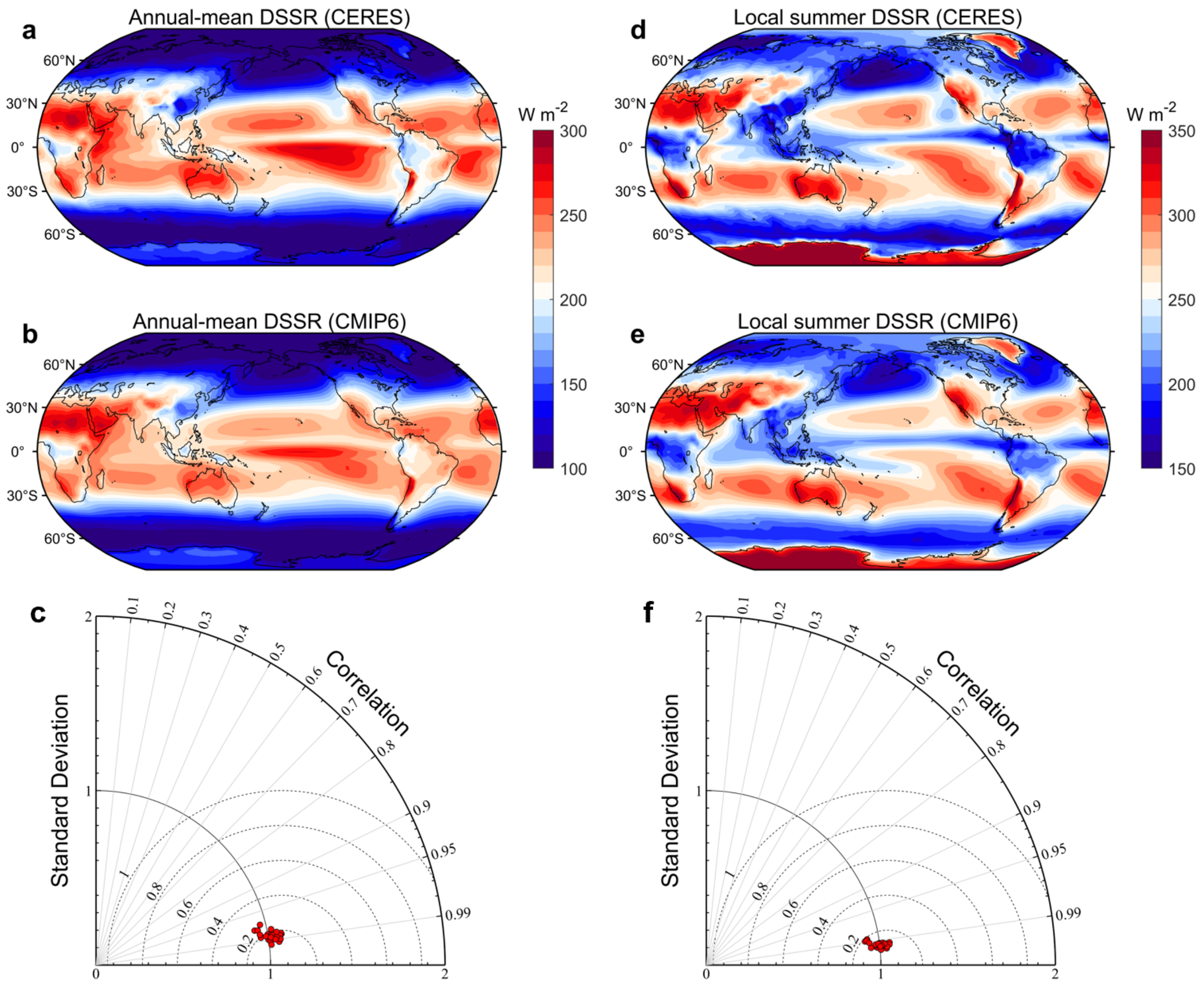

Fig. S1 Evaluation of CMIP6 Models in Simulating DSSR patterns. a, **b** Annual-mean all-sky DSSR climatology for 2001-2024 from (**a**) CERES and (**b**) MME of 36 CMIP6 models. **c**, Taylor diagrams comparing the annual-mean all-sky DSSR climatology of CMIP6 models with CERES, with each dot representing one model. **d-f** As in **a-c**, but for the local summer. Units in **a**, **b**, **d**, **e**: W m^{-2}

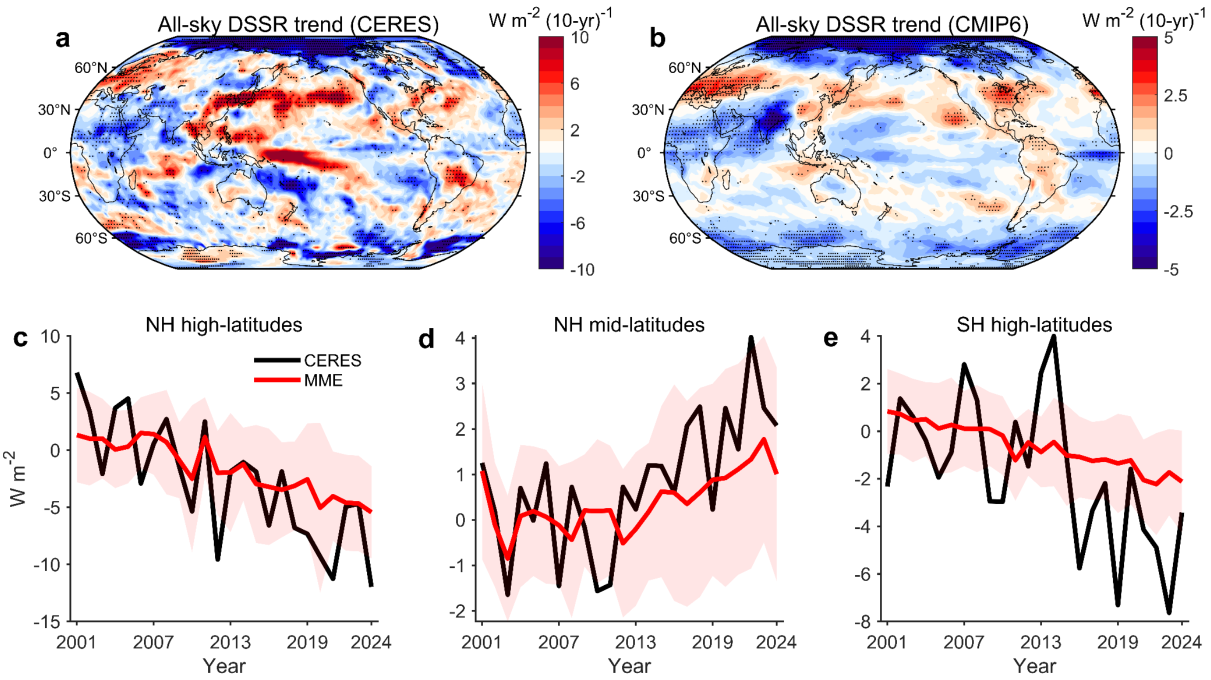

Fig. S2 Evaluation of CMIP6 Models in Simulating DSSR trends. **a**, **b** Spatial pattern of linear trends (unit: W m^{-2} (10-yr)$^{-}$1) of local summer all-sky DSSR during 2001-2024 in (**a**) CERES and (**b**) MME of 36 CMIP6 models. Stippling indicates regions where at least 70% of the models agree on the sign of the MME change. **c**-**e**, Time evolution of local summer all-sky DSSR in CERES (black) and MME (red) over (**c**) NH high-latitudes (70-90°N), (**d**) NH mid-latitudes (30-60°N), and (**e**) SH high-latitudes (60-90°S). Shadings mark the one standard deviation model spread. The reference period is 2001-2014. Units in **c**-**e**: W m^{-2}

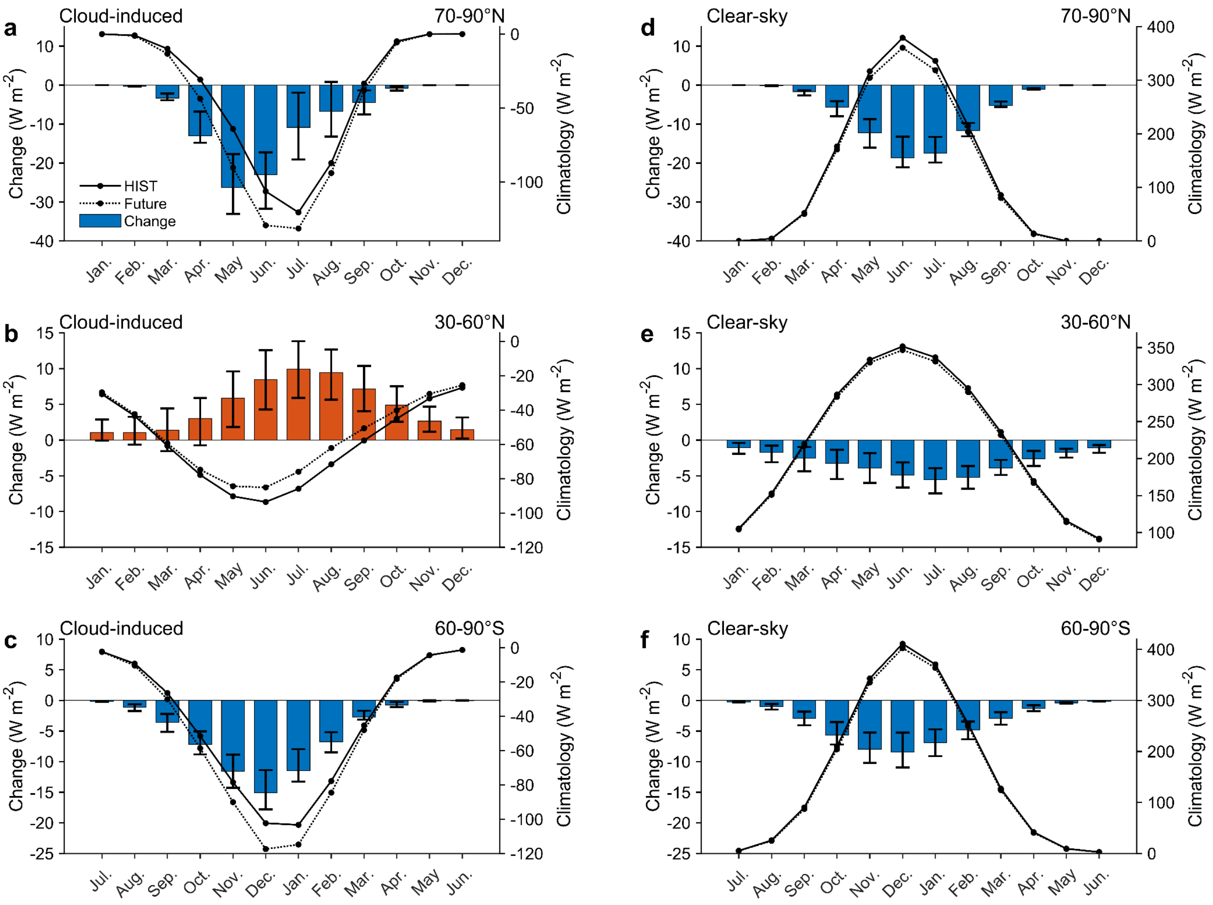

Fig. S3 Seasonal cycle changes in the two components of DSSR. a-c MME changes in cloud-induced DSSR of seasonal cycle at the (**a**) Arctic (70-90°N), (**b**) NH mid-latitudes (30-60°N), and (**c**) Antarctic (60-90°S). **d-f**, As for **a-c**, but for the clear-sky DSSR. Solid and dashed lines indicate the historical and future climatology, respectively. Bars show the MME monthly mean changes, and error bars denote the interquartile range (25th-75th percentile) of the model ensemble. Units: W m^{-2}

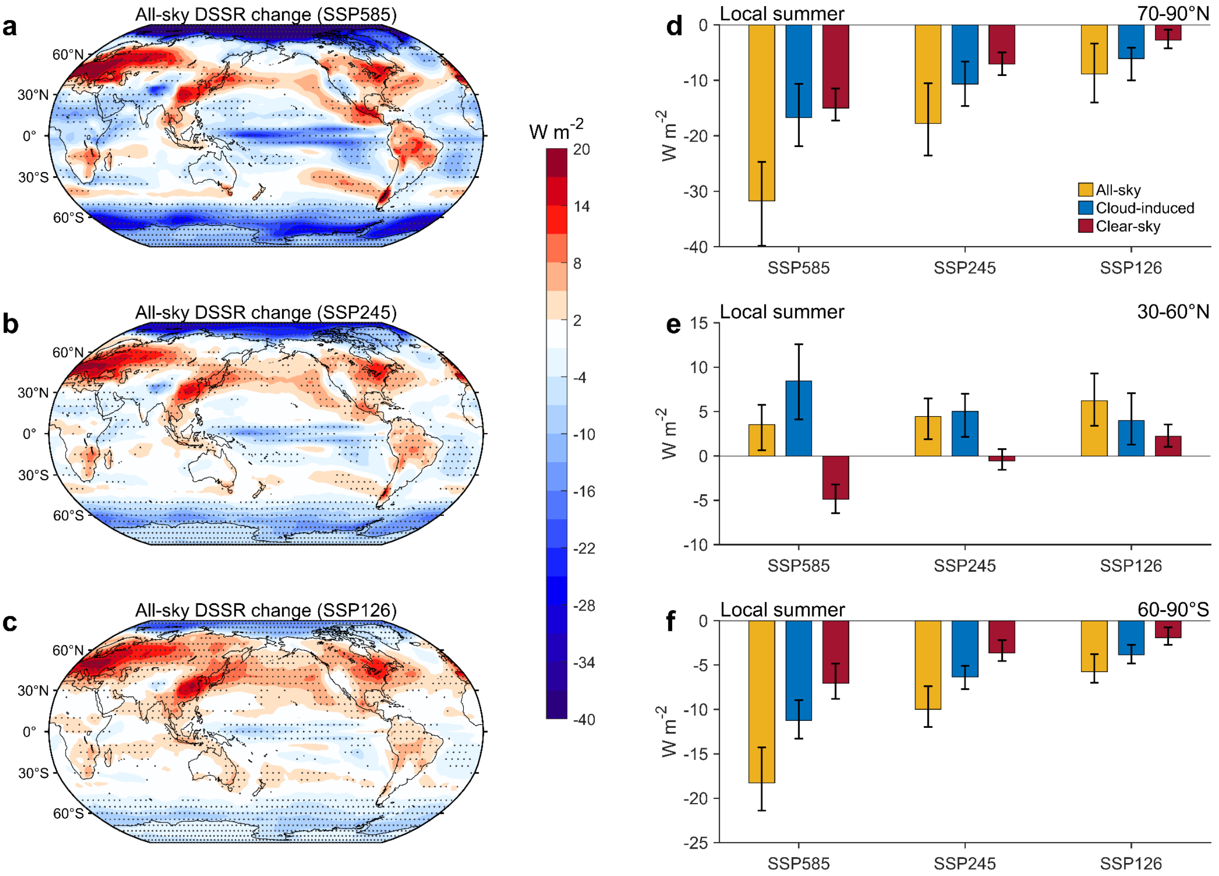

Fig. S4 DSSR changes under different emission scenarios. a-c Changes in all-sky downward surface solar radiation (DSSR) in the local summer (May-August (MJJA) for the NH and November-February (NDJF) for the SH) between 2080-2099 and 1986-2005 based on the multi-model ensemble (MME) of CMIP6 models under the (**a**) SSP585 high-emission, (**b**) SSP245 medium-emission, and (**c**) SSP126 low-emission scenarios, respectively. Stippling in a-c indicates regions where at least 70% of the models agree on the sign of the MME change. d-f MME changes in all-sky DSSR and its two components in the local summer at the (**d**) Arctic (70-90°N), (**e**) NH mid-latitudes (30-60°N), and (**f**) Antarctic (60-90°S) under different emission scenarios. Error bars denote the interquartile range (25th-75th percentile) of the model ensemble. Units in **a-f**: W m^{-2}.

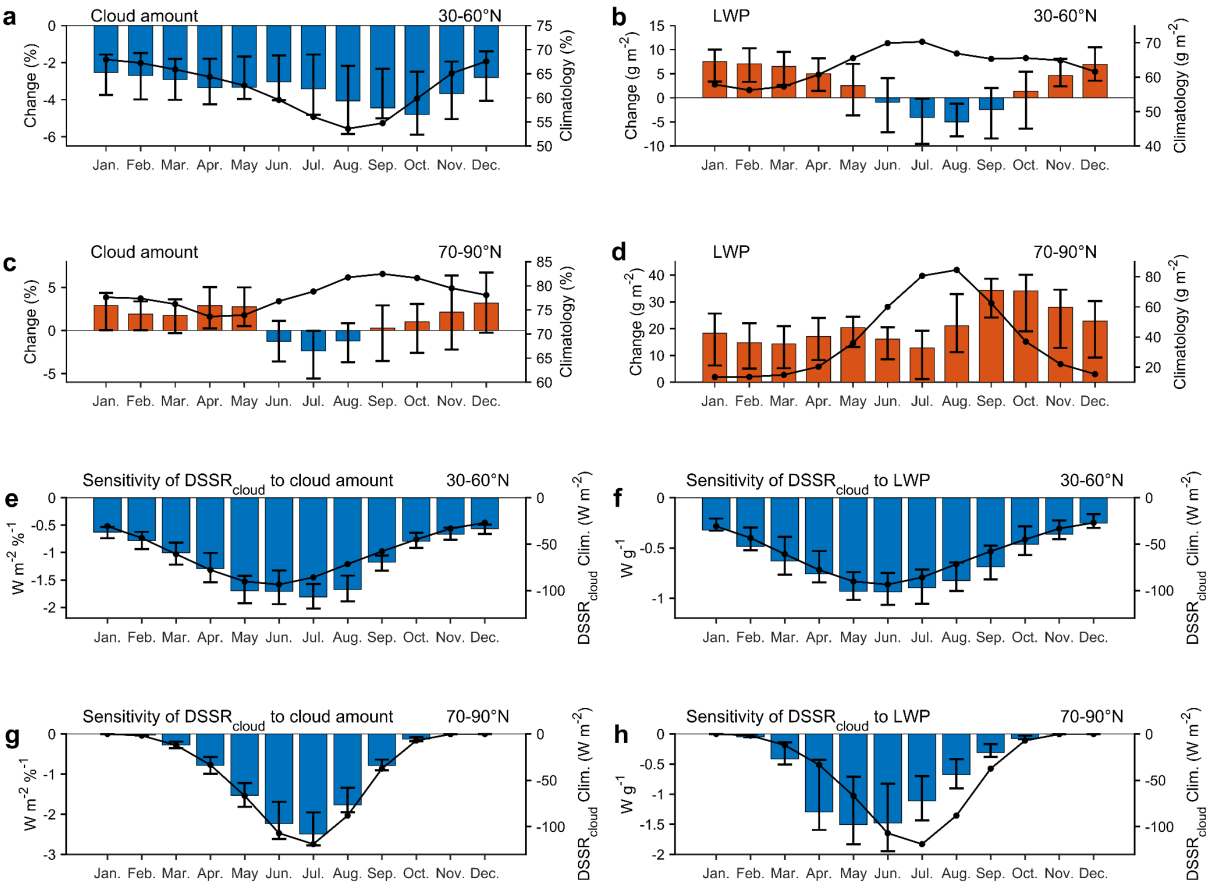

Fig. S5 Most pronounced summer cloud-induced DSSR changes are related to higher sensitivity. a, **b** Seasonal MME changes in (**a**) cloud amount and (**b**) LWP over the NH mid-latitudes (30-60°N). **c, d**, As in **a**, **b**, but for the Arctic. Solid lines indicate the historical climatology. Bars show the MME monthly mean changes, and error bars denote the interquartile range (25th-75th percentile) of the model ensemble. **e**, **f** Sensitivity of cloud-induced DSSR to cloud amount (unit: W m^{-2} $\%^{-1}$) over the (**e**) NH mid-latitudes and (**f**) Arctic for 1956-2005, based on the 36 CMIP6 models. Solid lines indicate the historical climatology of cloud-induced DSSR (unit: W m^{-2}). **g**, **h**, As in **e**, **f** but for the sensitivity to LWP (unit: W g^{-1}).

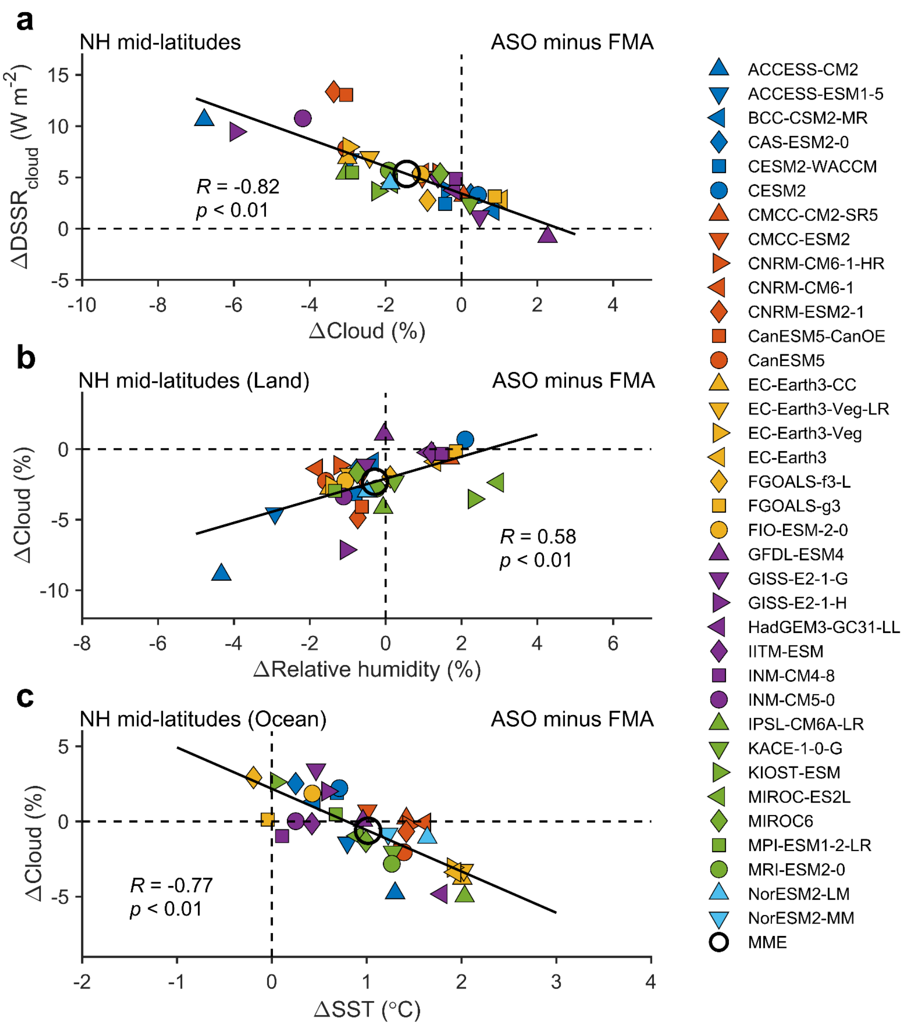

Fig. S6 NH mid-latitude DSSR seasonal delay due to the cloud amount change. a, Scatter plots of the inter-model relationship between changes in inter-seasonal differences (ASO minus FMA) in cloud amount change (unit: %) and cloud-induced DSSR change (unit: W m^{-2}) over NH mid-latitudes. **b**, **c**, As in **a**, but for the relationships between inter-seasonal changes in relative humidity (unit: %) and cloud amount over NH mid-latitude land (**b**), and between SST (unit: °C) and cloud amount over NH mid-latitude oceans (**c**). Linear fits, correlation coefficients (R), and p-values are shown.

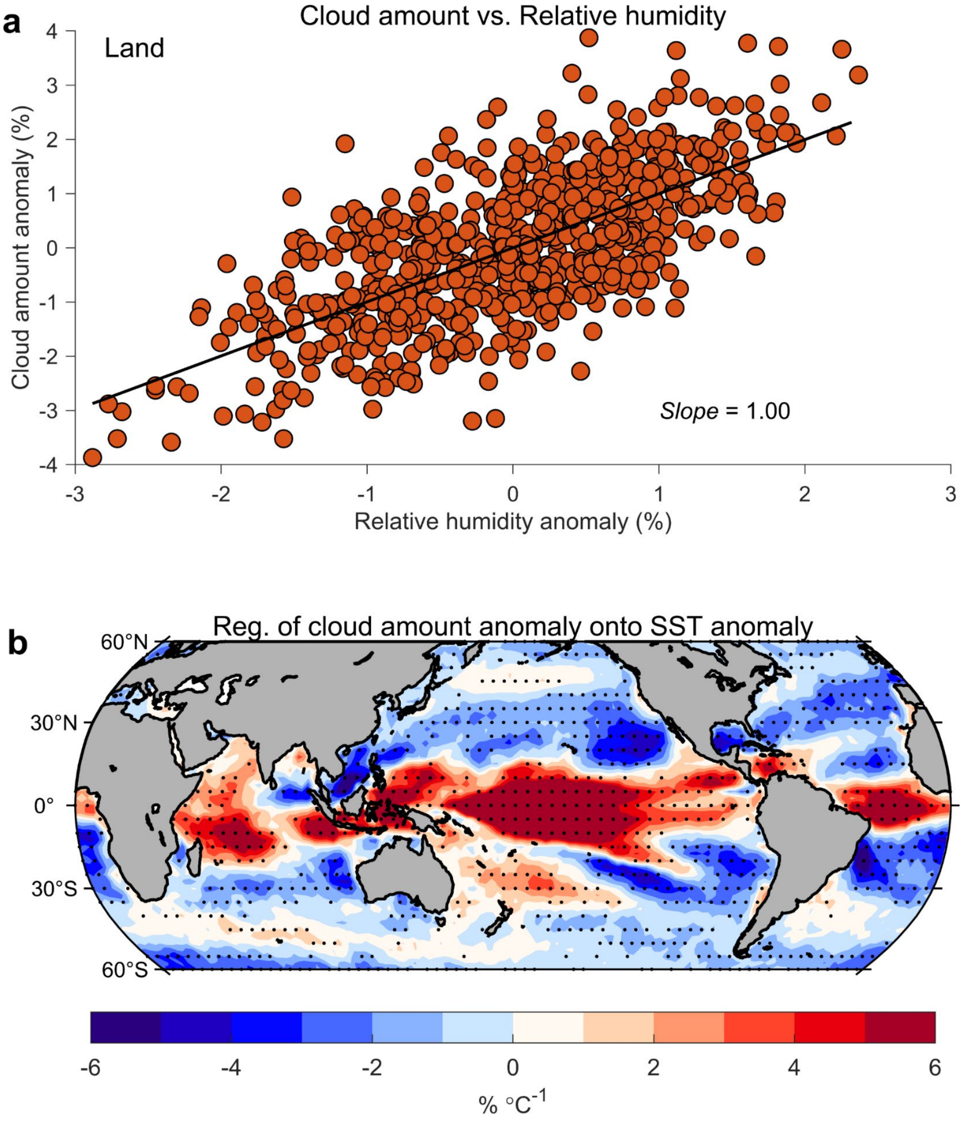

Fig. S7 Relationship between cloud amount and relative humidity and SST. a, Scatter plot between monthly anomalies of surface relative humidity (unit: %) and cloud amount (unit: %) over land (60°S–60°N). Each dot represents one month. The linear regression line and its slope are shown. **b**, Spatial distribution of the regression coefficient of monthly cloud amount anomalies onto monthly SST anomalies. Stippling indicates the coefficient is significantly above a 99% confidence level. Data in **a** and **b** are taken from the ERA5 reanalysis covering 1956–2005.

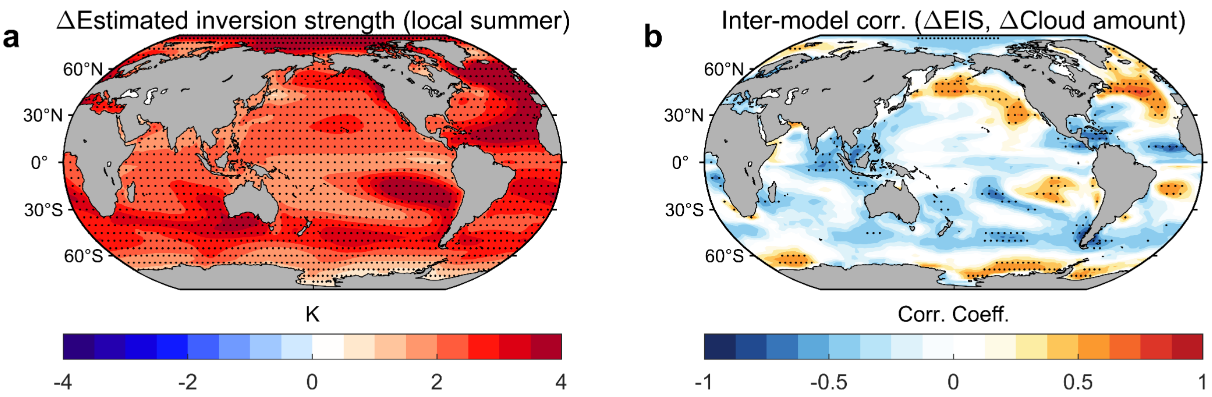

Fig. S8 Changes in estimated inversion strength. **a**, MME changes in estimated inversion strength (unit: K) for the local summer between 2080-2099 and 1986-2005. Stippling indicates regions where at least 70% of the models agree on the sign of the MME change. **b**, Spatial distribution of the inter-model correlation coefficient between the local summer changes in estimated inversion strength and cloud amount. Stippling indicates the coefficient is significantly above a 99% confidence level.

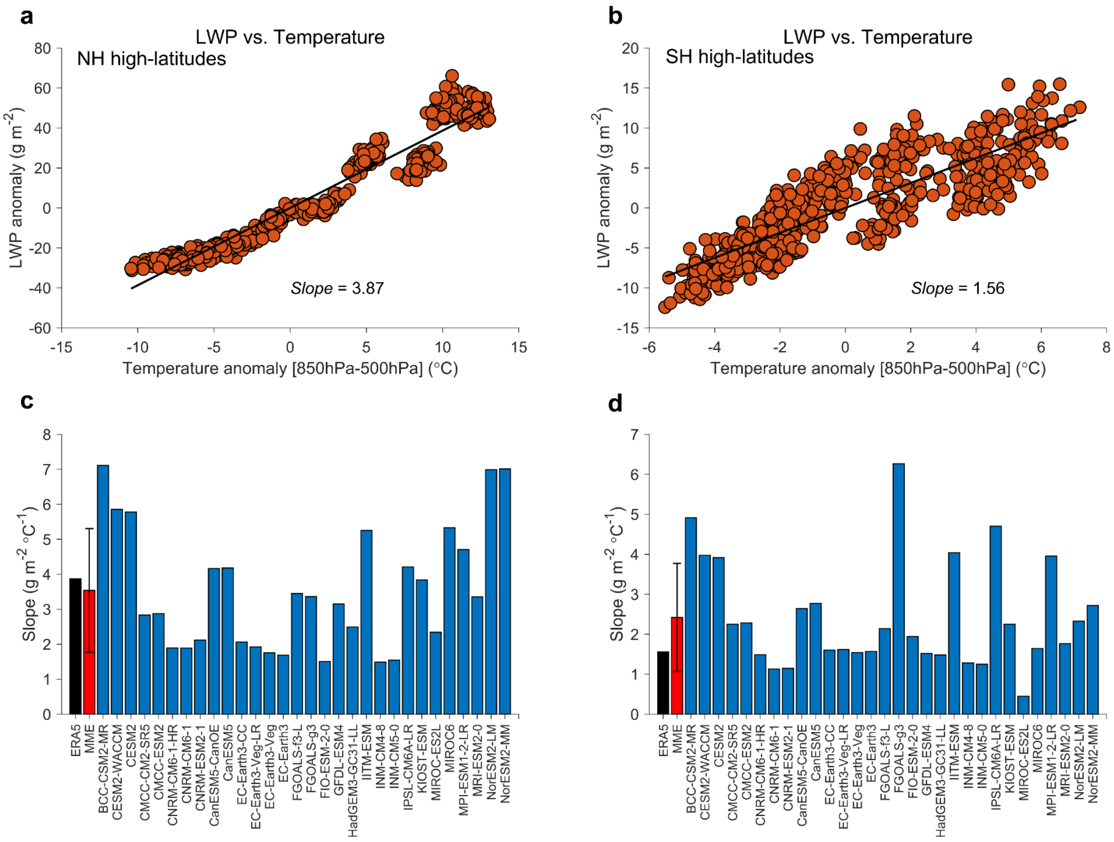

Fig. S9 Relationship between temperature and LWP. a, **b** Scatter plots of monthly anomalies of lower-tropospheric temperature (averaged between 850 and 500 hPa; unit: °C) versus LWP over (**a**) Arctic (70°N-90°N) and (**b**) Antarctic (60°S-90°S), respectively. Linear fits and slopes are shown. Data in **a** and **b** are derived from the ERA5 reanalysis for 1956-2005. **c**, **d** Comparison of the slopes (unit: g m^{-2} $°C^{-1}$) between ERA5 and CMIP6 models over (**c**) NH high latitudes and (**d**) SH high latitudes, with black, red, and blue bars representing ERA5, MME, and individual models, respectively. Error bar denotes the ±1 standard deviation across models.

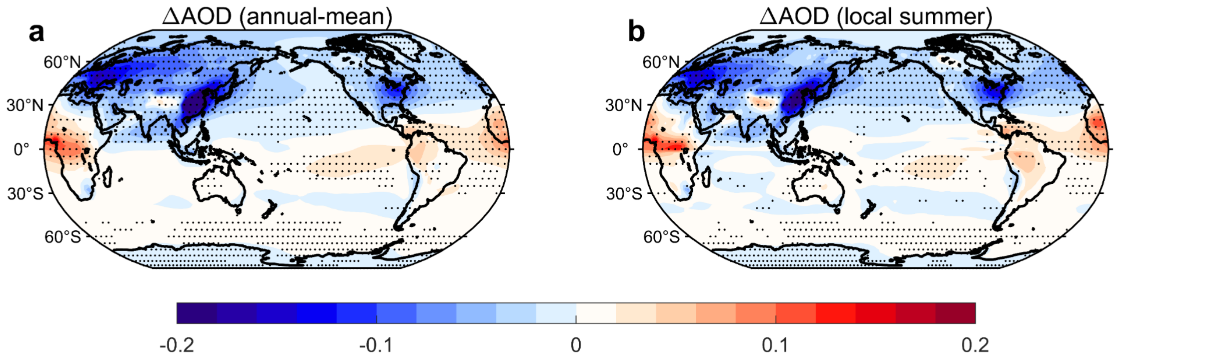

Fig. S10 Changes in AOD. a, **b** MME changes in optical thickness of atmospheric aerosols at wavelength 550 nm (AOD) for (**a**) the annual-mean and (**b**) local summer between 2080-2099 and 1986-2005. Stippling indicates regions where at least 70% of the models agree on the sign of the MME change.

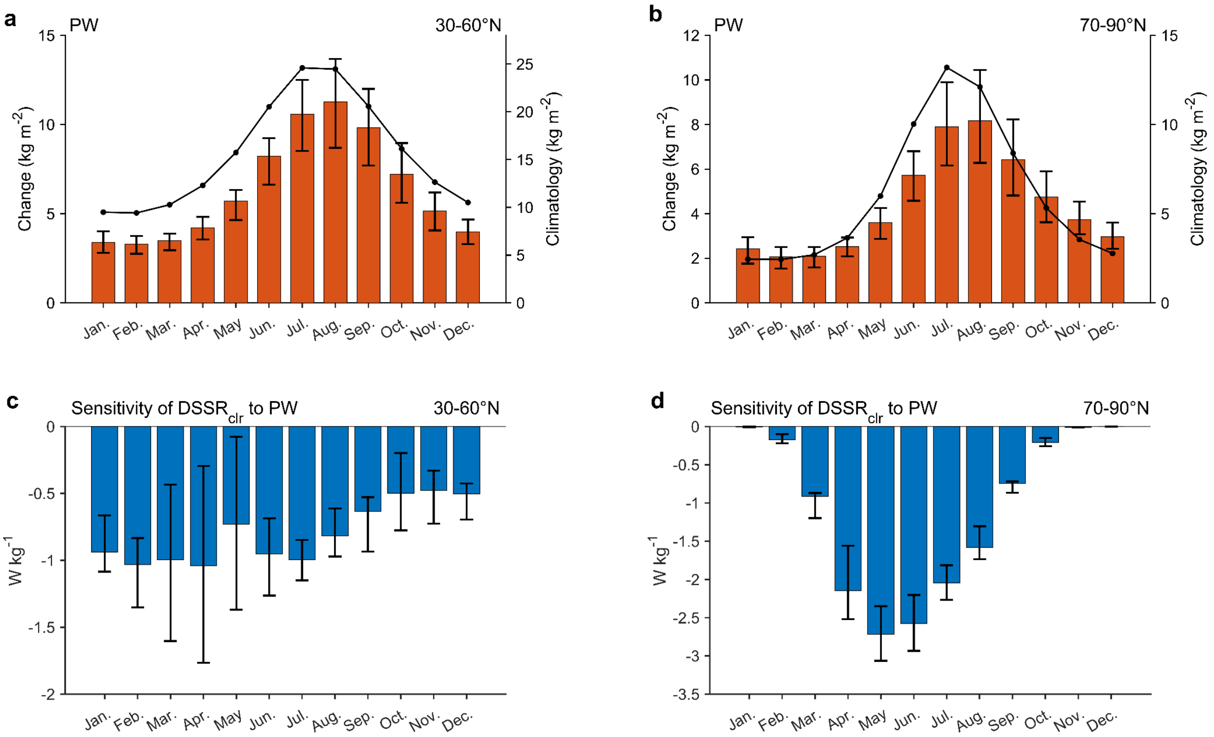

Fig. S11 Changes in the seasonal cycle of water vapor and the sensitivity of clear-sky DSSR to water vapor. **a**, **b** Seasonal MME changes in PW (unit: kg m^{-2}) (**a**) over the NH mid-latitudes (30-60°N) and (**b**) over the Arctic (70-90°N), respectively. Solid lines indicate the historical climatology. **c**, **d** Sensitivity of clear-sky DSSR to PW (unit: W kg^{-1}) over (**c**) the NH mid-latitudes and (**d**) Arctic for 1980-2020, based on the hist-GHG experiment of 11 CMIP6 models. Bars show the MME monthly mean value, and error bars denote the interquartile range (25th-75th percentile) of the model ensemble.

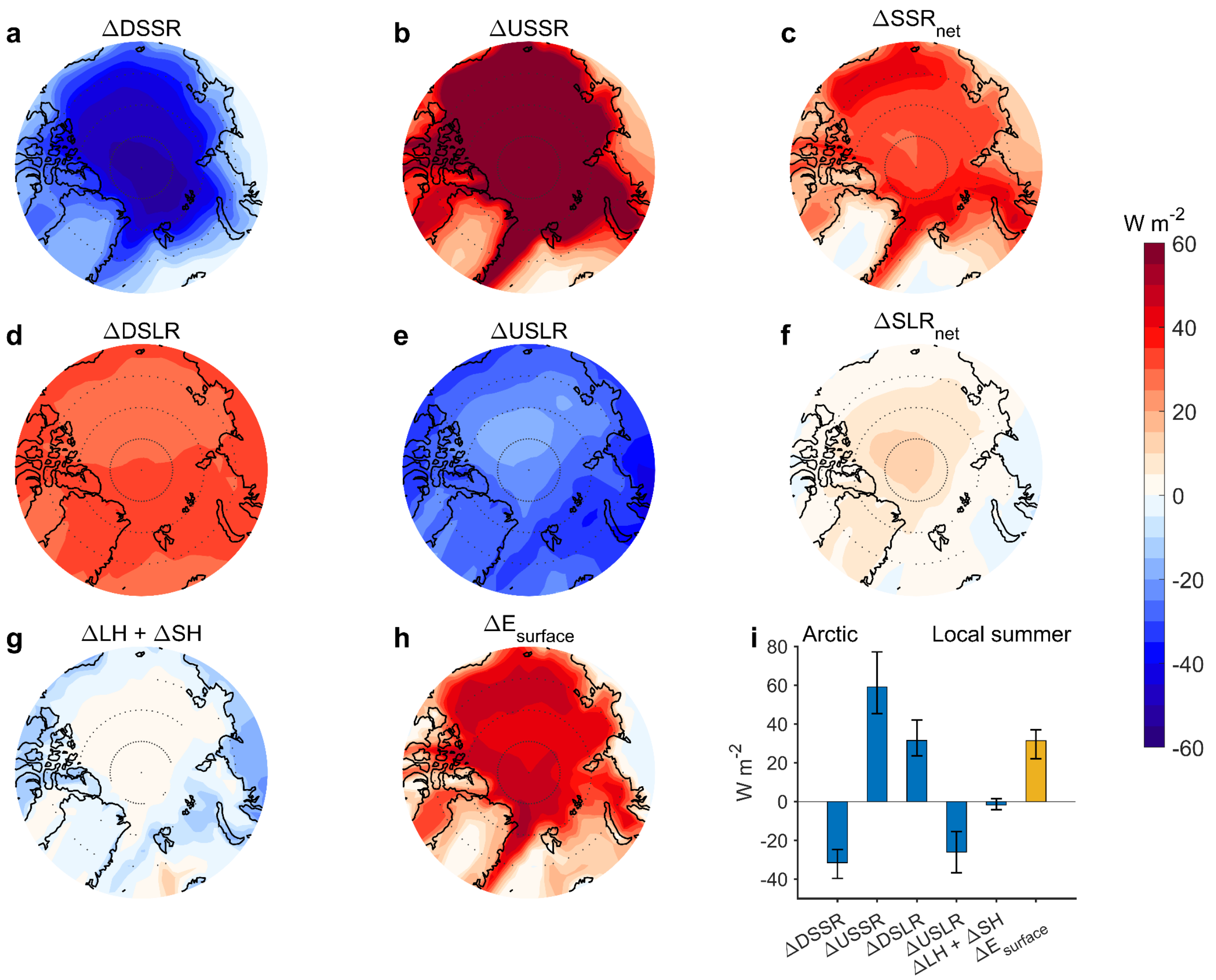

Fig. S12 Surface heat flux changes. Changes in (**a**) all-sky downward surface solar radiation (DSSR) for local summer (May-August (MJJA) for the NH and November-February (NDJF) for the SH) between 2080-2099 and 1986-2005 based on the multi-model ensemble (MME) of CMIP6 models. Stippling indicates regions where at least 70% of the models agree on the sign of the MME change. **b-h**, As in a, but for (**b**) upward surface solar radiation (USSR), (**c**) net surface solar radiation (SSR_{net}), (**d**) downward surface longwave radiation (DSLR), (**e**) upward surface longwave radiation (USLR), (**f**) net surface longwave (SLR_{net}), (**g**) latent heat plus sensible heat flux (LH+SH) and (**h**) net surface heat flux ($E_{surface}$). **i**, Changes in surface heat fluxes over the Arctic (70-90°N). Error bars denote the interquartile range (25th-75th percentile) of the model ensemble. All heat fluxes are defined as positive downward. All heat fluxes are defined as positive downward. Units: W m^{-2}

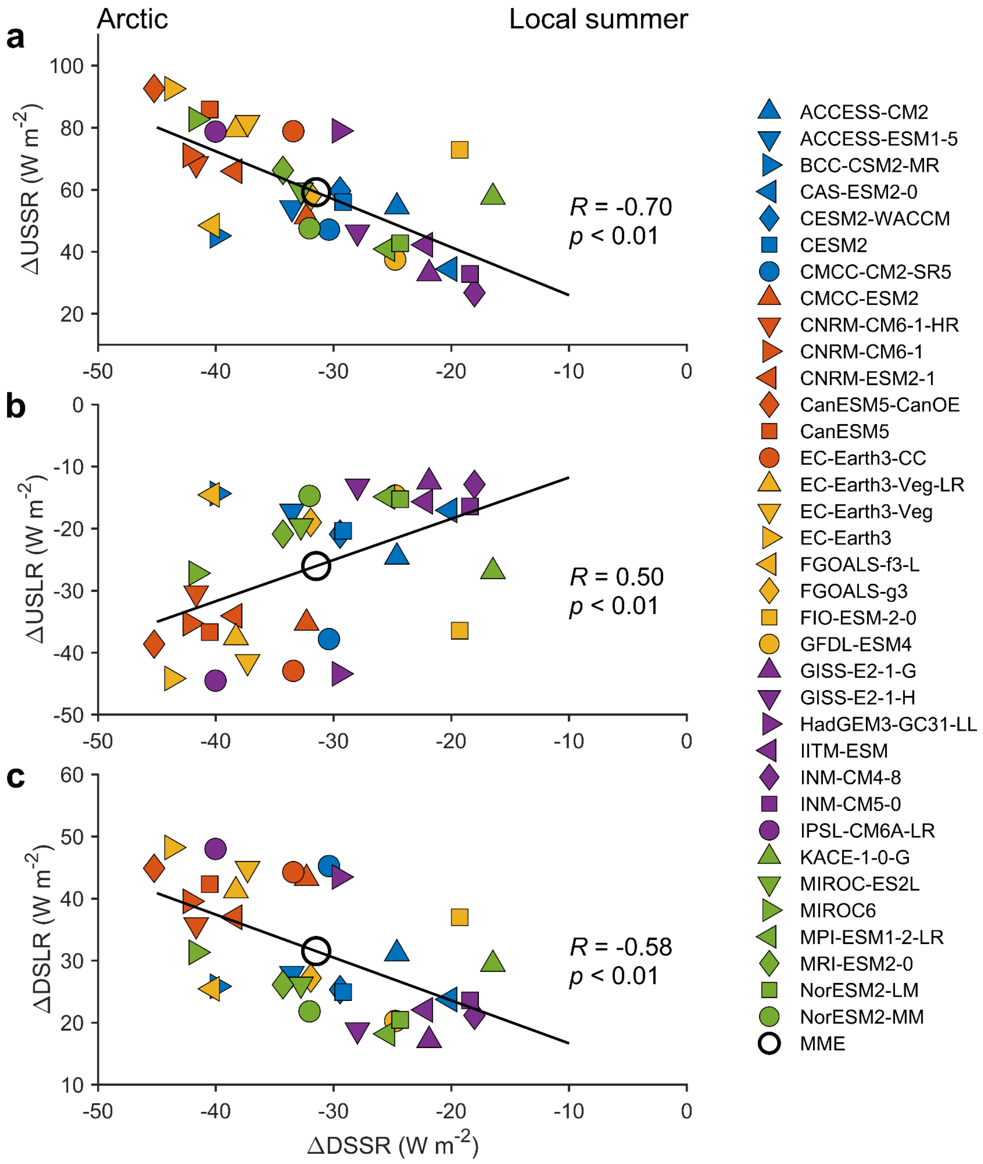

Fig. S13 Relationship between DSSR and other surface radiation fluxes. a, Scatter plots of the inter-model relationship between changes in DSSR and upward surface solar radiation (USSR) over the Arctic. **b**, **c**, As in **a**, but for the relationships between changes in DSSR and (**b**) upward surface longwave radiation (USLR) and (**c**) downward surface longwave radiation (DSLR), respectively. Linear fits, correlation coefficients (*R*), and *p*-values are shown. Units: W m^{-2}

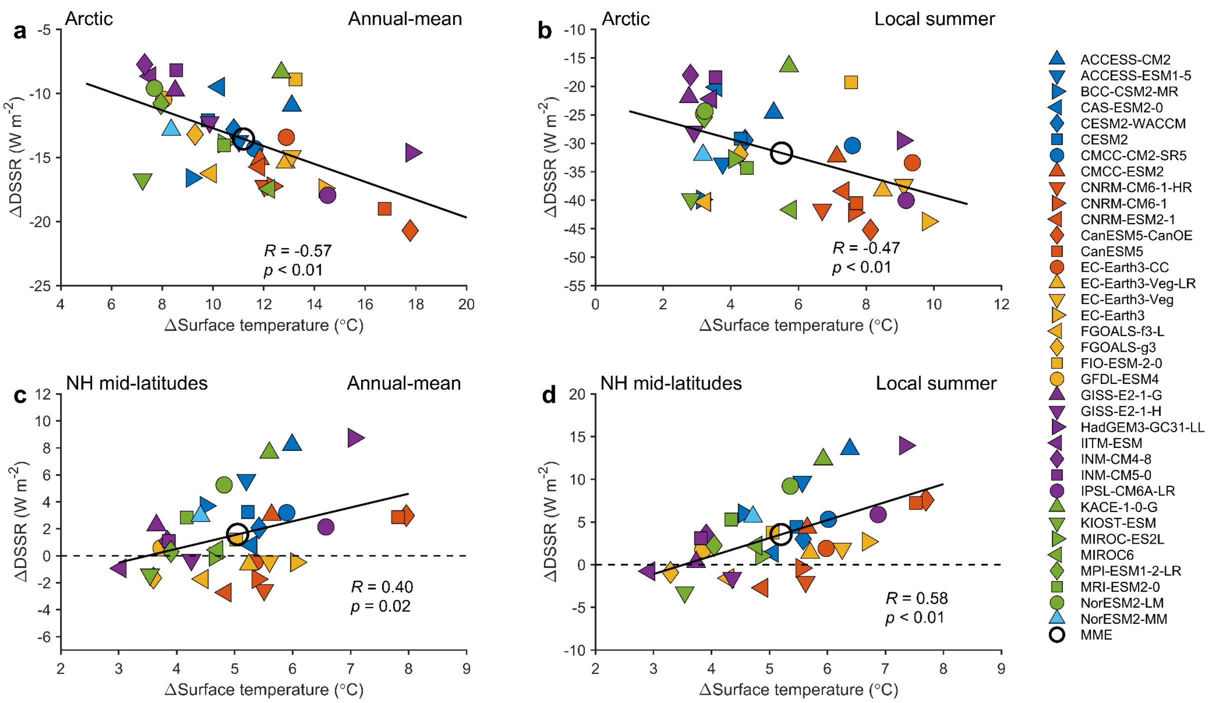

Fig. S14 Inter-model relationship between the meridional warming gradient and the DSSR changes. **a**, **b** Scatter plots of inter-model relationship between surface temperature changes (unit: °C) and the DSSR changes (unit: W m^{-2}) over the Arctic for (**a**) annual-mean and (**b**) local summer. Linear fits, correlation coefficients (R), and p-values are shown. **c**, **d,** As in **a**, **b**, but for the NH mid-latitudes (30°-60°N).

Table S1. The CMIP6 model variables used in this study.

Model Name	Variables	Label
ACCESS-CM2*	1,2,3,4,7,8,9,10,11,12,13,14	r1i1p1f1
ACCESS-ESM1-5*	1,2,3,4,7,8,9,10,11,12,13,14	r1i1p1f1
BCC-CSM2-MR*	1,2,3,4,5,6,8,9,11,12,13,14	r1i1p1f1
CanESM5*	1,2,3,4,5,6,7,8,9,10,11,12,13,14	r1i1p1f1
CanESM5-CanOE	1,2,3,4,5,6,7,8,9,10,11,12,13,14	r1i1p2f1
CAS-ESM2-0	1,2,3,4,5,6,7,8,9,11,12,13,14	r3i1p1f1
CESM2	1,2,3,4,7,8,9,10,11,12,13,14	r4i1p1f1
CESM2-WACCM	1,2,3,4,5,6,7,8,9,10,11,12,13,14	r1i1p1f1
CMCC-CM2-SR5	1,2,3,4,5,6,7,8,9,10,11,12,13,14	r1i1p1f1
CMCC-ESM2	1,2,3,4,5,6,7,8,9,10,11,12,13,14	r1i1p1f1
CNRM-CM6-1*	1,2,3,4,5,6,7,8,9,10,11,12,13,14	r1i1p1f2
CNRM-CM6-1-HR	1,2,3,4,5,6,7,8,9,10,11,12,13,14	r1i1p1f2
CNRM-ESM2-1	1,2,3,4,5,6,7,8,9,10,11,12,13,14	r2i1p1f1
EC-Earth3	1,2,3,4,5,6,7,8,9,10,11,12,13,14	r1i1p1f1
EC-Earth3-CC	1,2,3,4,5,6,7,8,9,11,12,13,14	r1i1p1f1
EC-Earth3-Veg	1,2,3,4,5,6,7,8,9,10,11,12,13,14	r2i1p1f1
EC-Earth3-Veg-LR	1,2,3,4,5,6,7,8,9,11,12,13,14	r1i1p1f1
FGOALS-f3-L	1,2,3,4,5,6,7,8,9,11,12,13,14	r1i1p1f1
FGOALS-g3*	1,2,3,4,5,6,7,8,9,11,12,13,14	r1i1p1f1
FIO-ESM-2-0	1,2,3,4,5,6,7,8,9,11,12,13,14	r1i1p1f1
GFDL-ESM4	1,2,3,4,5,6,7,8,9,10,11,12,13,14	r1i1p1f1
GISS-E2-1-G	1,2,3,4,7,8,9,11,12,13,14	r1i1p1f2

GISS-E2-1-H	1,2,3,4,7,8,9,11,12,13,14	r1i1p1f2
HadGEM3-GC31-LL*	1,2,3,4,5,6,7,8,9,10,11,12,13,14	r1i1p1f3
IITM-ESM	1,2,3,4,5,6,7,8,9,11,12,13,14	r1i1p1f1
INM-CM4-8	1,2,3,4,5,6,7,8,9,10,11,12,13,14	r1i1p1f1
INM-CM5-0	1,2,3,4,5,6,7,8,9,10,11,12,13,14	r1i1p1f1
IPSL-CM6A-LR*	1,2,3,4,5,6,7,8,9,10,11,12,13,14	r1i1p1f1
KACE-1-0-G	1,2,3,4,7,8,9,10,11,12,13,14	r1i1p1f1
KIOST-ESM	1,2,3,4,5,6,7,8,9,11,12	r1i1p1f1
MIROC6*	1,2,3,4,5,6,7,8,9,10,11,12,13,14	r1i1p1f1
MIROC-ES2L	1,2,3,4,5,6,7,8,9,10,11,12,13,14	r2i1p1f2
MPI-ESM1-2-LR	1,2,3,4,5,6,7,8,9,10,11,12,13,14	r1i1p1f1
MRI-ESM2-0*	1,2,3,4,5,6,7,8,9,10,11,12,13,14	r1i1p1f1
NorESM2-LM*	1,2,3,4,5,6,7,8,9,10,11,12,13,14	r1i1p1f1
NorESM2-MM	1,2,3,4,5,6,7,8,9,10,11,12,13,14	r1i1p1f1

"1" denotes surface downwelling shortwave radiation (all-sky DSSR), "2" denotes surface downwelling clear-sky shortwave radiation (clear-DSSR), "3" denotes surface upwelling clear-sky shortwave radiation, "4" denotes total cloud cover percentage, "5" denotes ice water path, "6" denotes condensed water path, "7" denotes near-surface relative humidity, "8" denotes surface air pressure, "9" denotes surface temperature, "10" denotes ambient aerosol optical thickness at 500 nm, "11" denotes air temperature, "12" denotes specific humidity, "13" denotes surface longwave radiation (upward and downward), and "14" denotes turbulent flux (latent and sensible heat). "*" denotes that the hist-GHG experiment of the model is used.

Each CMIP6 model identifier consists of four components: r, i, p, and f. The r denotes the realization number, where r1 indicates the first realization of the model run; The i denotes the initialization number, with i1 indicating the first initialization instance; The p denotes the physical parameterization scheme, where p1 represents the first physical configuration; Finally,

the f indicates the forcing scenario, with f1 representing the first forcing configuration.
